# Common and differential transcriptional responses to different models of traumatic stress exposure in rats

**DOI:** 10.1038/s41398-018-0223-6

**Published:** 2018-08-23

**Authors:** Moriah L. Jacobson, Lydia A. Kim, Robert Patro, Barbara Rosati, David McKinnon

**Affiliations:** 1 Department of Psychology, Stony Brook University, Stony Brook, NY 11794 USA; 2 Department of Neurobiology and Behavior, Stony Brook University, Stony Brook, NY 11794 USA; 3 Department of Computer Science, Stony Brook University, Stony Brook, NY 11794 USA; 4 Department of Physiology and Biophysics, Stony Brook University, Stony Brook, NY 11794 USA; 5 Institute of Molecular Cardiology, Stony Brook University, Stony Brook, NY 11794 USA; 6 Department of Veterans Affairs Medical Center, Northport, NY 11768 USA

## Abstract

The effect of six different traumatic stress protocols on the transcriptome of the rat adrenal gland was examined using RNA sequencing. These protocols included chronic variable stress, chronic shock, social defeat and social isolation. The response of the transcriptome to stress suggested that there are genes that respond in a universal or stress modality-independent manner, as well as genes that respond in a stress modality-specific manner. Using a small number of the genes selected from the modality-independent set of stress-sensitive genes, a sensitive and robust measure of chronic stress exposure was developed. This stress-sensitive gene expression (SSGE) index could detect chronic traumatic stress exposure in a wide range of different stress models in a manner that was relatively independent of the modality of stress exposure and that paralleled the intensity of stress exposure in a dose-dependent manner. This measure could reliably distinguish control and stressed individuals in the case of animals exposed to the most intense stress protocols. The response of a subset of the modality-specific genes could also distinguish some types of stress exposure, based solely on changes in the pattern of gene expression. The results suggest that it is possible to develop diagnostic measures of traumatic stress exposure based solely on changes in the level of expression of a relatively small number of genes.

## Introduction

The original description of the stress response by Selye^1^ emphasized the nonspecific nature of the response to a range of different stressors. Subsequently, it was shown that, at least in some tissues, the response to different modalities of stress exposure can be distinguished^2,3^. Distinct hormonal and neurotransmitter release responses to different stressors have been observed following exposure to a variety of physical stressors^2^. Additionally, different neural pathways are activated by either physical or psychological stressors^3^. That the response to stress can contain both common and mode-specific elements rather than being an invariant response to different modalities of stress is consistent with the complexity of the neural, hormonal and gene regulatory networks that underlie the stress response^4–9^.

To date, relatively little is known about how the gene regulatory network responds to widely varying forms of traumatic stress and whether or not there are distinct transcriptional responses to different forms of stress. The adrenal gland is an important tissue in which to study this question. The somatic response to stress is mediated via the hypothalamic–pituitary–adrenal axis (HPA) and the autonomic nervous system. The adrenal gland is the terminal organ for both the HPA axis and the sympathetic adrenomedullary (SAM) system^10,11^. It is, therefore, a key tissue in which to ask basic questions about the gene regulatory response to stress, because changes in gene expression in this tissue are likely to be strongly tied to mediating the responses to stress. In addition, there is interest in developing biological diagnostic tests for stress exposure and any such test will most likely use peripheral molecules/cells as a substrate for testing. The adrenal gland mediates a large fraction of the peripheral responses to stress and understanding the response of this tissue is of general interest for solving this translational problem.

In this study, the response of the adrenal gland transcriptome to six different chronic stress protocols of varying modality and intensity was examined. RNA sequencing was used for the transcriptome analysis, facilitating detection of both common and differential responses to the different stress modalities. The stress models were designed to test two hypotheses: (i) that gene expression profiles can distinguish animals exposed to traumatic stress from controls, and (ii) that there are quantifiable differences in the response of the transcriptome to different models of stress exposure. The results show clear differences in the response of the adrenal gland to different stress protocols, as measured by changes in gene expression. Nonetheless, a set of stress modality-independent responses, which may reflect a universal transcriptional response to chronic stress in this tissue, were also observed. These results have translational relevance in that they demonstrate that it is possible to diagnostically distinguish individuals exposed to traumatic stress from controls, as well as distinguish types of stress exposure solely on the basis of changes in the pattern of gene expression.

## Results

### Diverse and common changes in response to intense chronic stress

A total of six different chronic stress paradigms were developed: social isolation, social defeat (SD), SD with social isolation, grid housing (GH), chronic shock (CS) and chronic variable stress (CVS) (see Methods for details). We initially focused on the two most intense protocols, CS and CVS, which use very different procedures to induce stress.

**Fig. 1:**
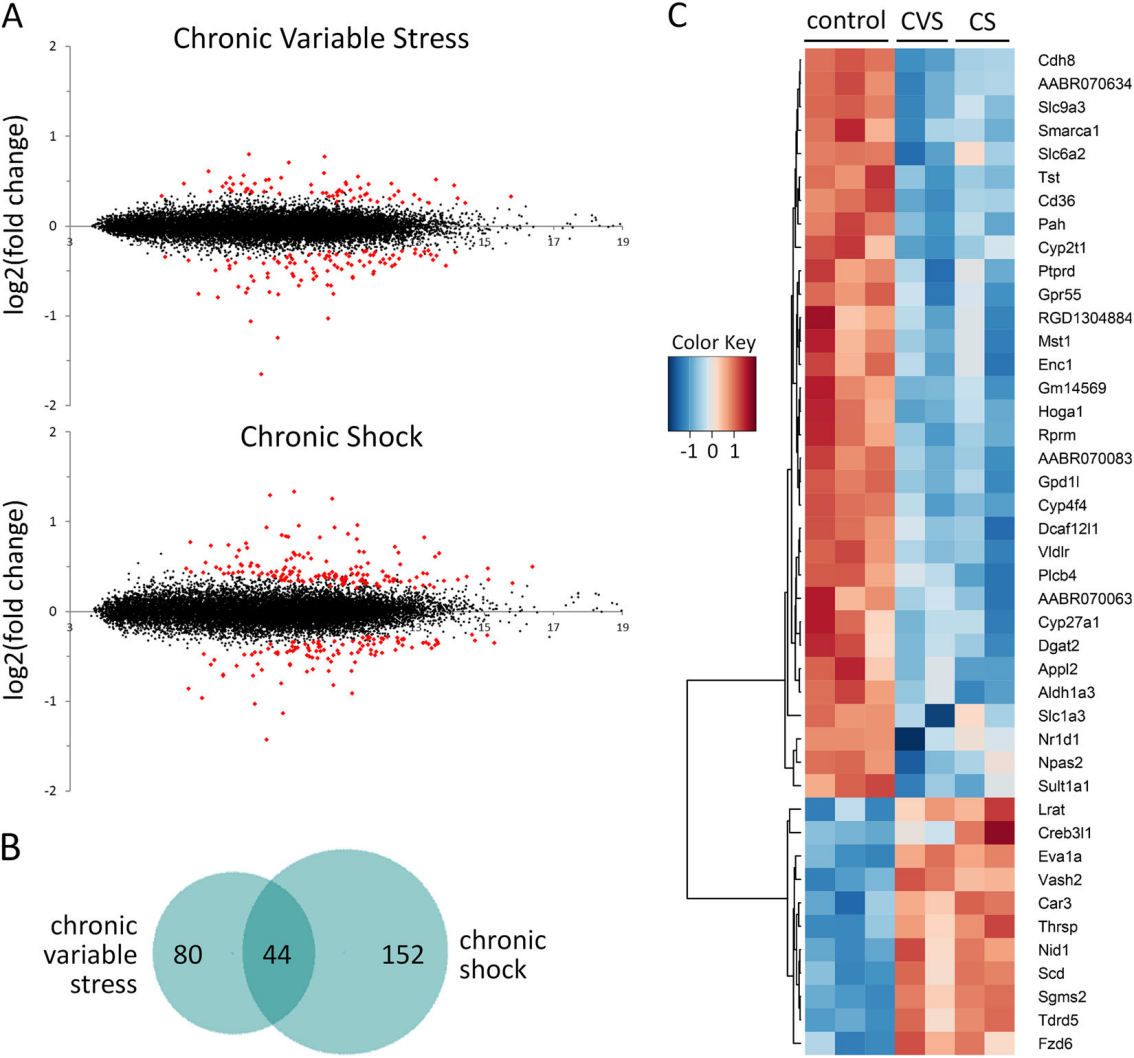
**Changes in Rat Adrenal Gland Transcriptome in Response to Chronic Variable Stress (CVS) or Chronic Shock (CS)**. **a** MA plots of RNA-sequencing data from adrenal gland for animals exposed to the chronic variable stress (CVS) or chronic shock (CS) protocols. The *x* axis corresponds to log2(average expression) and the *y* axis to log2(Stress/Control). Differentially expressed genes (marked in red) were selected using FDR = 0.05. RNA samples were pooled (*n* = 8–9) before sequence analysis for each of the four independent replicates of the experimental groups (2 CVS and 2 CS) and three independent control groups. Fold-change values reflect dispersion moderation in DESeq2. See Tables S2 and S3 for complete list of differentially expressed genes. **b** Venn diagram of the differentially expressed genes for the two different stress groups. The total number of differentially expressed genes was 124 for the CVS protocol, 196 for CS protocol and 44 genes were differentially expressed in both protocols. **c** Heat map for those genes changed in both CVS and CS stress protocols. Expression values were log2 transformed and scaled to each row

In the adrenal gland, a relatively large number of genes were found to be differentially expressed following exposure to each of these protocols (Fig. 1a). There were more changes in response to the CS protocol than to the CVS protocol, with 196 genes differentially expressed following the CS protocol versus 124 genes following the CVS protocol (False Discovery Rate (FDR) = 0.05). Notably, a majority of the genes that changed in response to CVS did not change in the CS animals and vice versa (Fig. 1b).

A subset of 44 genes was found to be differentially expressed in both protocols (Fig. 1b, c, and Table S1). With a few exceptions, this common set of differentially expressed genes changed similarly in response to both stress protocols (Fig. 1c). A preponderance of these genes were downregulated.

Gene ontology (GO) analysis showed that a similar set of GO terms were over- or under-represented for both the CS and CVS groups. A total of 46 GO terms were differentially represented for the CS group compared with 29 terms for the CVS group (Tables S4 and S5). More terms were identified for the CS group, as expected given the larger number of genes found to be differentially expressed in this protocol. All the terms identified for the CVS group were also on the CS list, suggesting considerable overlap in terms of the effect of the two stress protocols on cellular function in the adrenal gland.

### Stress-sensitive gene expression index

The existence of a common set of differentially expressed genes, which respond to both of these very different stress protocols, suggested that these genes might be universal markers of traumatic stress exposure in the adrenal gland. We hypothesized that a subset of these common response genes could be used to construct a stress-sensitive gene expression (SSGE) index that could be used to measure stress exposure in a consistent and quantitative way across a broad array of chronic stress modalities. It was anticipated that this index might function in an analogous way to a stock market index, which can capture the mood of the market by sampling only a small subset of the most characteristic stocks.

Genes were selected from the common set of 44 genes (Table S1) for potential inclusion in the SSGE index based on several criteria. Only protein coding genes with the largest effect size in Table S1 were considered. In addition, only genes for which the effect size was reasonably similar in both the CS and CVS protocols (see Tables S2 and S3) were selected. As a final criterion, quantitative PCR (qPCR) was used to determine the level of variation in gene expression between individual samples within the control groups. Genes with relatively stable levels of expression in the controls were favored for inclusion in the index. Based on these criteria, six genes were selected: *Pah*, *Slc9a3*, *Thrsp*, *Scd*, *Cdh8* and *Cd36*. The index was deliberately restricted to a relatively small number of genes in order to simplify measurement across large numbers of individual animals.

**Fig. 2:**
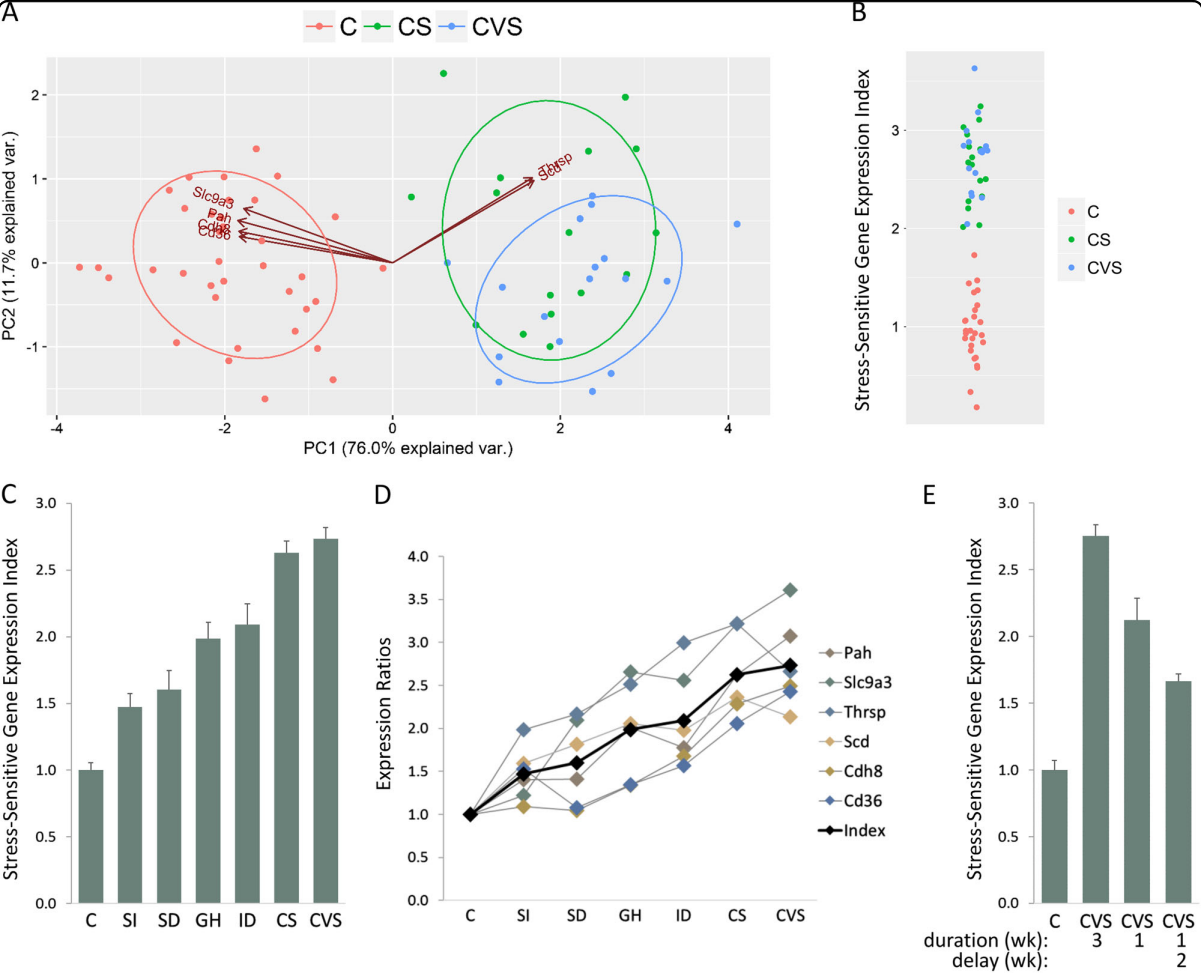
**Measurement of Stress Exposure with a Stress Sensitive Gene Expression (SSGE) Index**. **a** Principal component analysis using qPCR expression values for six genes (*Pah*, *Slc9a3*, *Thrsp*, *Scd*, *Cdh8* and *Cd36*) comparing control (*n* = 36) animals and animals exposed to the two most intense stress protocols (*n* = 33), either chronic variable stress (CVS) or chronic shock stress (CS). **b** Strip plot comparing the stress-sensitive gene expression (SSGE) index for control animals and animals exposed to either chronic variable stress (CVS) or chronic shock stress (CS). **c** Average SSGE index for control (C) and six different stress groups: social isolation (SI), social defeat (SD), grid housing (GH), isolation defeat (ID), chronic variable stress (CVS) and chronic shock (CS). There was a difference between the groups as determined by one-way ANOVA (*F*(6, 105) = 55.97, *p* < 2 × 10^−16^). All stress groups were different to the control group: SI (*p* = 1 × 10^−4^, *d* = 1.2), SD (*p* = 1 × 10^−4^, *d* = 1.5), GH (*p* = 1 × 10^−8^, *d* = 2.8), ID (*p* = 6 × 10^−10^, *d* = 2.8), CS (*p* < 2 × 10^−16^, d = 4.5) and CVS (*p* < 2 × 10^−16^, *d* = 4.9). **d** Mean expression ratios for each of the six selected genes compared with the index (black) across the seven different experimental groups. **e** Comparison of SSGE index for control (C), the standard 3-week duration CVS protocol (CVS), a protocol of 1-week CVS and a protocol of 1-week CVS followed by 2 weeks of social housing. There was a difference between the groups as determined by one-way ANOVA (*F*(3, 48) = 76.5, *p* < 2 × 10^−16^). All stress groups were different to the control group: CVS (*p* < 2 × 10^−16^, *d* = 5.3), CVS 1 week (*p* = 2 × 10^−9^, *d* = 2.9) and CVS 1 week with 2 week delay (*p* = 6 × 10^−3^, *d* = 2.7). Error bars are s.e.m

Principal component analysis (PCA) of the expression values derived from qPCR analysis of gene expression for the six genes selected for the index demonstrated that there was no overlap between individuals in the stress (CVS or CS) and control groups along the axis of the first principal component (Fig. 2a). This is important because it implies that these expression values can be used to reliably distinguish individuals exposed to chronic stress from controls.

A SSGE index was then calculated as follows. For each individual, *j*, an expression ratio, $x_j^i$, was determined for each gene, *i*,

$$\begin{array} {ccc}x_j^i =&& \log _2\left( {C^i/y_j^i} \right) + 1:\,{\mathrm{for}}\,{\mathrm{down}} {\mathrm{regulated}}\,{\mathrm{genes}}\,Pah, \cr &&Slc9a3,Cdh8\,{\mathrm{and}}\,Cd36\end{array}$$

$$\begin{array} {ccc}x_j^i =&& {\mathrm{log}}_2\left( {y_j^i/C^i} \right) + 1:\,{\mathrm{for}}\,{\mathrm{up}}{\mathrm{regulated}}\,{\mathrm{genes}}\cr &&\left ( {Thrsp\,{\mathrm{and}}\,Scd} \right)\end{array}$$

where $y_j^i$ was the mRNA expression value for gene, *i*, in individual, *j*, and *C*^*i*^ was the mean expression level for gene, *i*, in the control animals. Expression ratios were converted to logarithm base 2 to maintain within-group variance relatively constant across the different experimental groups. The expression ratios, $x_j^i$, were normalized so that the mean, $x_j^i$, for the control samples, for each gene, *i*, was equal to unity. Expression ratios $x_j^i$ for each of the genes were then averaged to give the SSGE index, *I*_j_, for each individual, *j* (worked example in Supplementary Information). The index has a mean value of unity for the control samples.

The SSGE index performed, as well as PCA, in distinguishing all the individual animals in the CVS and CS groups from the control animals (Fig. 2b). The advantage of the index over PCA was that the index scale is only dependent on the average expression values in the control samples. These average values were stable across experimental replicates, facilitating the comparison of stress exposure across different protocols.

### SSGE index applied to multiple stress protocols

To determine whether the index could perform in a predictable manner across different modalities of stress exposure, average index values were determined for the control group and the six different stress protocol groups (Fig. 2c). There was a difference between the groups as determined by one-way analysis of variance (ANOVA) (*F*(6, 105) = 55.97, *p* < 2 × 10^−16^) and all stress groups were different to the control group (Fig. 2c).

Based on body and organ weight changes (see below), the CVS and CS stress protocols were more stressful than the other protocols and were similar in intensity to each other. This assessment was also reflected in the stress index results (Fig. 2c). The average SSGE index values increased in a manner consistent with the relative intensity of the different stress protocols. Social isolation was the least stressful of these protocols but exposure to this protocol could still be readily detected, with the SSGE index being higher in the stress group than controls, who were socially housed (*p* = 1 × 10^−4^, *d* = 1.2). It is reasonable to assume that GH would be more stressful than social isolation because it combines social isolation with difficult housing conditions. Indeed, the SSGE index was found to be higher for GH in comparison with the social isolation group (*p* = 3 × 10^−3^, *d* = 1.3). Similarly, it is reasonable to assume that CS would be more stressful than GH, because this adds electric shocks to the social isolation and difficult housing conditions. As expected, the index was higher for this group relative to GH (*p* = 5 × 10^−4^, *d* = 1.8). The relatively high score for GH was consistent with our observations of the rat’s general condition and the lengths to which the animals would go to get off the grid, even in the absence of shock.

Rats are less likely to display symptoms of anxiety following SD when they are housed socially rather than in isolation^12^, suggesting that the isolation defeat (ID) is more stressful than SD. The difference in the respective index values for the two protocols (*p* = 0.01, *d* = 1.1) was consistent with this expectation.

Although the individual expression ratios for each of the six genes that contribute to the index responded somewhat differently to the different stress protocols, the trend for each expression ratio across the six different protocols was in broad agreement with the overall trend of the SSGE index (Fig. 2d). For the three most intense protocols (ID, CS and CVS), expression levels of all six individual genes were differentially expressed relative to the controls (*p* < 0.005). These genes can reasonably be considered universally responsive to these different stress modalities. The number of genes that were differentially expressed relative to controls fell as the protocols became less intense (GH: 5/6, SD: 3/6, SI: 3/6), suggesting that some genes in the index are more responsive to lower levels of stress than others.

The performance of the index was not particularly sensitive to the number or combination of genes included in the index. Reducing the number of index genes to only four, those with the largest effect size (*Pah*, *Slc9a3*, *Thrsp* and *Scd*), did not substantially alter the performance of the index, although it did result in a modest increase in the within-group variance (average S.D. for each group with six index genes was 0.39 versus 0.49 with only four genes).

To examine the effect of different durations of stress exposure, the standard CVS protocol (3-week duration) was compared with a shorter CVS protocol (1-week duration) (Fig. 2e). The magnitude of the SSGE index after the 1-week stress exposure was smaller than that seen for the 3-week protocol (*p* = 1 × 10^−4^, *d* = −1.5), but remained well above control levels (*p* = 2 × 10^−9^, *d* = 2.9), suggesting that shorter periods of stress exposure can also be readily detected by the index. The time period over which the SSGE index remains elevated following the cessation of stress exposure was examined by exposing animals to a 1-week duration CVS protocol and then returning them to social housing for 2 weeks before analysis. In this case, the SSGE index remained elevated compared with controls (*p* = 6 × 10^−3^, *d* = 2.7) although the index value was smaller than for the other two CVS groups, where stress exposure occurred closer to the time of measurement (Fig. 2e). This result suggests that the index decays relatively slowly, with a time period of weeks.

### Weight changes in response to chronic stress

**Fig. 3:**
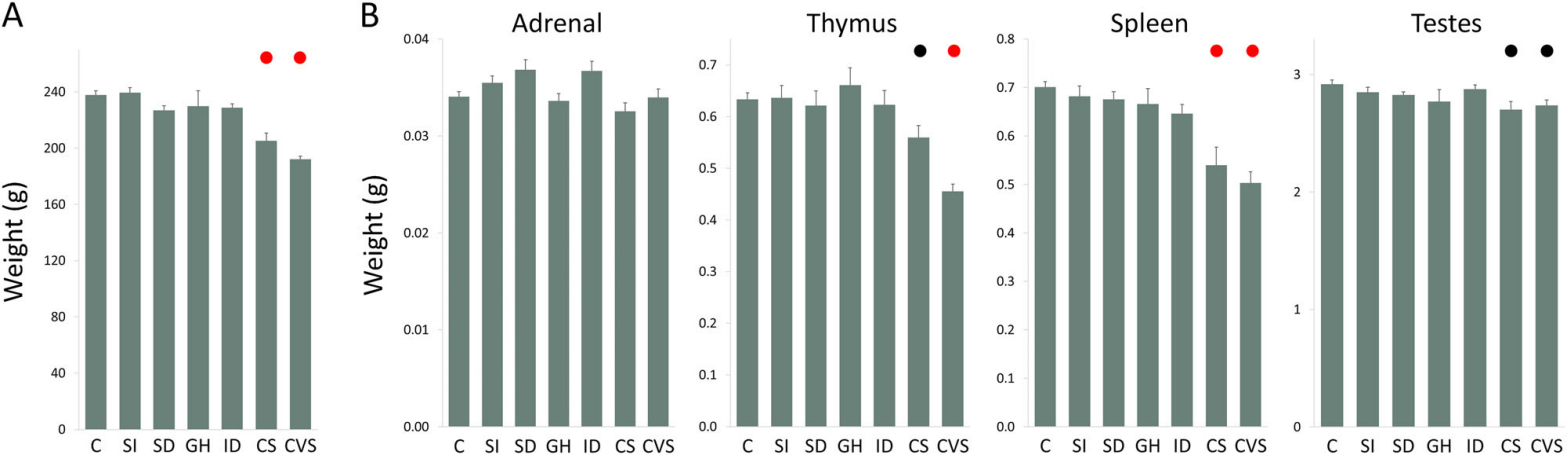
**Effects of Chronic Stress on Whole Body and Organ Weights in Rats**. **a** Body weight 1 day after the end of the stress protocols in control and chronically stressed animals. There was a difference between the groups as determined by one-way ANOVA (*F*(6, 105) = 18.0, *p* = 3 × 10^−14^). Average weights for stress groups that were changed relative to the control group are marked with colored circles, *p* < 0.05 (black) and *p* < 0.001 (red). **b** Organ weights for adrenal gland, thymus, spleen and testes. One-way ANOVA for: adrenal gland (*F*(6, 105) = 2.95, *p* = 0.01), thymus (*F*(6, 105) = 11.2, *p* = 1 × 10^−9^), spleen (*F*(6, 70) = 11.5, *p* = 6 × 10^−9^) and testis (*F*(6, 105) = 2.78, *p* = 0.02). Error bars are s.e.m

Reduction in body weight is a common response to chronic stress exposure^13^. Changes in body and organ weight in response to the different stress protocols were consistent with the results from the SSGE index suggesting that the CS and CVS protocols were more intense than the other stress protocols (Fig. 3). Average body weight was reduced relative to controls 1 day after the end of the 3-week stress exposure period for both the CS and CVS groups (*p* = 1 × 10^−7^, *d* = −1.6; and *p* = 1 × 10^−12^, *d* = −3.3; respectively) (Fig. 3a).

Adrenal gland weight was not changed in any of the stress protocols relative to controls (Fig. 3b). Thymus weight was reduced relative to controls for the CVS group (*p* = 2 × 10^−9^, *d* = −2.7) with a smaller effect for the CS group (*p* = 0.01, *d* = −0.9). Thymus weight was lower in the CVS group than in the CS group (*p* = 2 × 10^−3^, *d* = −1.3). Spleen weight was reduced for both the CS (*p* = 4 × 10^−6^, *d* = −1.9) and CVS (*p* = 5 × 10^−8^, *d* = −3.2) groups. Testis weight was modestly reduced for both the CS and CVS groups (*p* = 0.02, *d* = −0.9; and *p* = 0.05, *d* = −0.9; respectively).

### Stress modality-specific gene expression

**Fig. 4:**
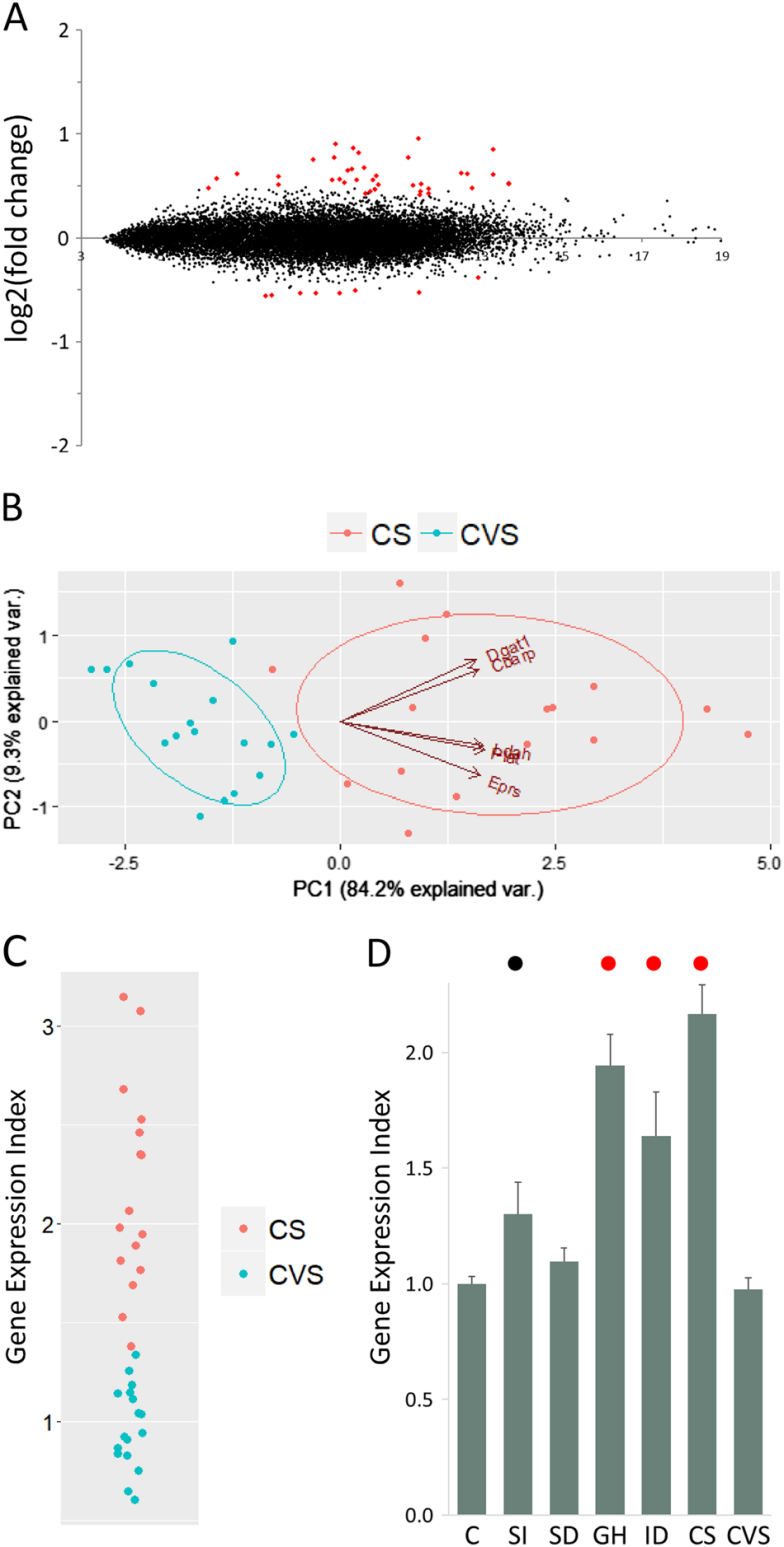
**Differential Gene Expression Responses to Chronic Variable Stress (CVS) or Chronic Shock (CS)**. **a** Comparison of RNA-sequencing counts from adrenal gland for animals exposed to the chronic variable stress (CVS) or chronic shock (CS) protocols. Differentially expressed genes (marked in red) were selected using FDR = 0.1. RNA samples were pooled (*n* = 8–9) before sequence analysis for each of the two independent biological replicates of the experimental groups. Fold-change values reflect dispersion moderation in DESeq2. **b** Principal component analysis for qPCR expression values for five genes (*Cbarp*, *Dgat1*, *Eprs*, *Ldah* and *Plat*) in two different stress exposure protocols: chronic variable stress (*n* = 17) and chronic shock (*n* = 16). **c** Strip plot comparing the gene expression index for animals exposed to either chronic variable stress (CVS) or chronic shock stress (CS). **d** Gene expression index using expression values for these same genes for control (C) and six different chronic stress groups: social isolation (SI), social defeat (SD), grid housing (GH), isolation defeat (ID), chronic shock (CS) and chronic variable stress (CVS). There was a difference between the groups as determined by one-way ANOVA (*F*(6, 105) = 23.5, *p* < 1 × 10^−17^). The average index value for each of the groups subject to isolation housing (SI, GH, ID and CS) was higher than the control group: SI (*p* = 0.01, *d* = 0.7), GH (*p* = 4 × 10^−8^, *d* = 3.2), ID (*p* = 1 × 10^−4^, *d* = 1.6) and CS (*p* = 1 × 10^−15^, *d* = 3.1). In contrast, the three social housing groups (C, SD and CVS) were not different to each other. Groups that were different to controls are marked on the histogram with colored circles, *p* < 0.05 (black) and *p* < 0.001 (red). Error bars are s.e.m

A direct comparison of the RNA-sequencing counts for the CS and CVS groups demonstrated that multiple genes were differentially expressed between the two stress groups (Fig. 4a). There was an excess of genes upregulated in the CS group relative to the CVS group. Five genes (*Cbarp*, *Dgat1*, *Eprs*, *Ldah* and *Plat*) were selected, based on effect size and consistent expression in controls, as assessed from qPCR data. PCA of the qPCR expression values for these genes suggested that they could distinguish most individuals in the two different stress treatment groups (Fig. 4b). An index based on the expression of these five genes was created, similarly to that described above for the SSGE index. As seen for the PCA, this index could distinguish the two stress groups relatively well (Fig. 4c).

When examined across all stress groups, this index had an interesting property. In addition to distinguishing the CVS and CS groups, it distinguished those groups where animals had been housed socially (C, SD and CVS) from those that had been housed in isolation (SI, GH, ID and CS) (Fig. 4d). This index did not solely reflect the effect of social isolation, two groups (GH and CS) that were subjected to additional stressors showed a further increment on this index relative to the SI group (*p* = 4 × 10^−4^, *d* = 1.3; and *p* = 2 × 10^−8^, *d* = 1.6; respectively). Nonetheless, the index was unexpectedly capable of identifying those groups that were singly housed.

### Behavioral responses to chronic stress

**Fig. 5:**
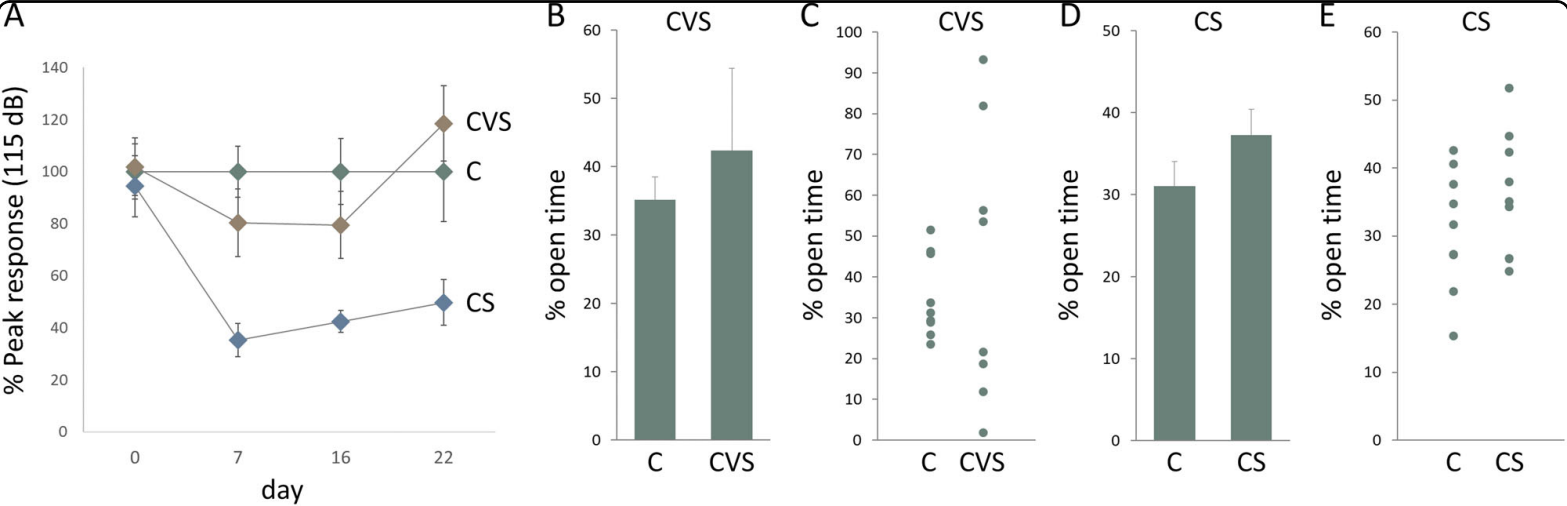
**Differential Behavioral Responses to Chronic Stress in the Acoustic Startle Response (ASR) and Elevated Plus Maze (EPM) Tests**. **a** Acoustic startle response (ASR). ASR tests were administered on days 0, 7, 16 and 22, relative to the start of the stress protocol on day 1. Results for control (C) (green symbol), chronic variable stress (CVS) (brown symbol) and chronic shock (CS) (blue symbol) are shown. The average startle response of the CS group was lower than the control group on days 7, 16 and 22 (*p* = 0.0004, *d* = −2.3; *p* = 0.002, *d* = −1.9; and *p* = 0.01, *d* = −1.4, respectively), whereas the response of the CVS groups was unchanged on any test day. Data were normalized so that the average peak response of the control group to a 115 dB pulse for each trial was equal to 100. Similar results were also seen using 95 and 105 dB test pulses. Data values are means with s.e.m. error bars (*n* = 8 or 9). **b** Elevated plus maze (EPM). Average time spent in the open arm as a percentage of the total time, for the control and CVS groups. Data values are means with s.e.m. error bars (*n* = 8 or 9). **c** Individual open time values for the same EPM experiment as shown in **b**. **d** Elevated plus maze (EPM). Average time spent in the open arm as a percentage of the total time, for the control and CS groups. Data values are means with s.e.m. error bars (*n* = 8 or 9). **e** Individual open time values for the same EPM experiment as shown in **d**

It has previously been reported that behavioral responses can be quite sensitive to the modality of stress exposure^14^. We examined two behavioral tests that are often used as measures of anxiety-like behavior, the acoustic startle response (ASR) and the elevated plus maze (EPM). The ASR was tested before the start of the stress protocol and then tested once a week for the following 3 weeks of stress exposure (Fig. 5a). Animals in the CVS and CS exposure groups responded quite differently in this test (Fig. 5a), despite the fact that both stress protocols appear to be similarly intense. CS exposure produced a persistent decline in the startle response, whereas CVS had no significant effect on the startle response.

Modality-specific effects were also seen using the EPM (Fig. 5b, c). There was no significant difference between the CVS and the control groups in the mean values for time spent in the open arms of the EPM (Fig. 4b) but there was a large increase in the variance for the CVS group (Fig. 5c). The CVS animals tended to ‘freeze’ in one of the compartments. Mean time spent in the ‘frozen’ state over the 5-min test period was much higher for the CVS animals (92 ± 34 s) than for controls (0.8 ± 0.6 s) (*p* = 0.01, *d* = 1.4). Freezing behavior was also reflected in an average 38% reduction in distance traveled by the CVS group compared with controls (*p* = 0.009, *d* = −1.5). In contrast, the CS protocol had no significant effect on the rats’ performance in the EPM (Fig. 5d, e).

## Discussion

The results described in this report demonstrate that there are both common and stress modality-specific transcriptional responses to different traumatic stress protocols in the adrenal gland. The common responses presumably reflect the fact that much of the stress response is funneled through the adrenal gland via a small number of signaling pathways^10,11^. The presence of an apparently universal response to widely varying stress modalities is consistent with the original description of the stress response as a nonspecific response^1^. Nonetheless, even in the adrenal gland, stress modality-specific responses are also observed, consistent with more modern concepts of the stress response having modality-specific elements^2,3^.

This study establishes a baseline for how much information is necessary to create a diagnostic test of traumatic stress exposure. We show that changes in gene expression can be used to create a quantitative and close-to-universal measure of stress exposure, and that sufficient information is encoded in the gene regulatory network to reliably distinguish unstressed from stressed subjects. This suggests that it is a practical possibility to use changes in gene regulatory function as a measure of traumatic stress exposure. Exactly the best way to exploit this information in humans remains uncertain but it is important to establish that the gene regulatory network encodes adequate information to make this distinction.

The SSGE index described here is a practical tool for quantitating levels of stress exposure in animal models. It is relatively easy and inexpensive to implement and many steps can be automated so that it can be scaled for large numbers of animals. There are several different ways to measure the effects of chronic stress exposure in laboratory animals^15^, including behavioral tests and analysis of endocrine function. Behavioral tests can be very sensitive but behavioral responses can also be strongly dependent on the modality of stress exposure^14^ (see Fig. 5). Endocrine responses to stress can be complex, transitory and can habituate in the maintained presence of the stressor^9,16,17^. They can also depend on stress modality^13^. In principle, the gene regulatory apparatus can integrate a variety of stress-related signals over time producing a more stable and robust signal for measurement.

The SSGE index appears to be as sensitive as most behavioral tests currently used to measure chronic stress exposure. Isolation housing is established as a stressor in rats^18–20^ but its effects are not always apparent using standard behavioral assays^12,21^ and results from different studies can be contradictory^20^. The SSGE index could clearly distinguish the social isolation group (rats housed singly) from the control group (rats housed three per cage) (Fig. 2c), suggesting that the assay is at least as sensitive as typical behavioral tests.

It could be asked, why are the genes selected for the SSGE, which were selected based on their sensitivity to two relatively intense stress protocols (CVS and CS), also sensitive to the much milder stressor social isolation? The underlying hypothesis of this study was that there would be genes that responded in a graded way in response to stress dosage and the data described in this report supports this initial hypothesis. This does not mean that all stress-sensitive genes will respond in a dose-dependent manner to stress or that such genes will necessarily respond to all modalities of stress. There appear to be a range of behaviors at the gene expression level, which is to be expected in such a large and complex regulatory network.

Any biological test of traumatic stress exposure in humans will most likely rely on peripheral tissues/molecules and thus be affected by stress-induced changes in adrenal gland function. The current study maps the global transcriptional response of the adrenal gland to different stress modalities. It is possible that markers in the bloodstream directly related to changes in adrenal cell gene expression described here will ultimately prove useful for a diagnostic test. The products of several of the genes in the index, including *Thrsp*, *Scd* and *Pah*, can be measured in the serum or plasma^22–24^, which could make them, or similar molecules, a practical pathway for testing. Alternatively, metabolites in the pathways in which these stress-sensitive genes function are a potential test substrate. The stress response of other peripheral tissues that can be more easily biopsied, such as white blood cells or buccal cells, will be strongly dependent on the response of the adrenal gland to stress. If these tissues ultimately prove useful for a diagnostic test of stress exposure in humans, it is of value to understand the extent and characteristics of the underlying behavior of the adrenal gland, which drives stress-dependent changes in these other tissues.

This work has translational relevance in two other respects. First, it identifies stress-responsive genes that may prove to be useful targets for blocking the peripheral stress response, particularly in chronic stress conditions were these responses can have long-term negative effects on health^25^. Second, the stress-responsive genes identified in this study may aid in the identification of susceptibility genes for trauma-related stress from genome-wide association studies.

In addition to the universal response genes, stress-responsive genes that were sensitive to the modality of stress exposure were also identified. This result suggests that studies using preclinical models should take account of the fact that the stress response, even at the level of transcription, can be sensitive to the modality of stress exposure and that validation of any potential stress-sensitive biomarker requires testing against multiple different models of stress exposure to ensure cross-modality validity. The differential responses to different modalities of stress did, however, suggest that it might be possible to distinguish the predominant nature of stress exposure using gene expression data, at least for some kinds of stress exposure. Such a test worked well for the comparison between the CS and CVS protocols. These were very different kinds of stress exposure, however, and whether subtler distinctions are possible remains unclear.

Many of the genes found to be differentially expressed in the adrenal gland following stress exposure in this study have previously been reported to be stress sensitive^26,27^. All of the genes included in the SSGE index (Table 1) have been implicated in the response to stress in one or more tissues^28–36^. Currently, however, relatively little is known about the specific role that these genes might play in mediating the response to stress in the adrenal gland. Phenylalanine hydroxylase (Pah) catalyzes the irreversible conversion of l-phenylalanine into l-tyrosine, an essential step in the catecholamine biosynthesis pathway^37^. However, it is generally believed that tyrosine synthesis occurs predominantly in the liver, with only the downstream catecholamine synthesis steps occurring in the adrenal medulla^10^. Three genes (*Thrsp*, *Scd* and *Cd36*) are involved in fatty acid metabolism. The adrenal gland has a high content of lipids, whose main function is to serve as precursors for steroid hormone biosynthesis in the cortex^38^. Changes in expression of genes implicated in lipid metabolism and transport in the adrenal gland may indicate a long-term effect of stress on steroid hormone biosynthesis. Expression of Cadherin-8 (Cdh8) is decreased in the prefrontal cortex in response to stress and it has been hypothesized that this may alter tissue plasticity to favor adaptive synapse remodeling^36^. The Na/H exchanger (Slc9a3) uses an inward sodium ion gradient to expel acids from the cell^39^. Its role in sodium and pH homeostasis has been studied mainly in the kidney and intestine and its function in adrenal gland tissue has not been characterized. These genes or their products are all potential targets for interventions designed to minimize the peripheral effects of stress.

**Table 1:** SSGE index genes and their potential role in stress

**Protein name**	**Gene symbol**	**Specific function**	**Potential role in stress response of adrenal gland**
Phenylalanine hydroxylase	Pah	Converts phenylalanine into tyrosine	Catecholamine biosynthesis
Sodium-hydrogen antiporter 3	Slc9a3	Imports one Na^+^ in the cell/exports one H^+^	Unknown
Thyroid hormone-responsive gene	Thrsp	Regulator of lipid metabolism	Altered fatty acid metabolism in adrenal gland
Stearoyl-CoA desaturase	Scd	Biosynthesis of monounsaturated fatty acids	Altered fatty acid metabolism in adrenal gland
Cadherin-8	Cdh8	Mediates cell–cell adhesion	Adaptive changes in tissue plasticity
Cluster of differentiation 36	Cd36	Fatty acid transport	Altered fatty acid metabolism in adrenal gland

Changes in gene expression in the adrenal gland could reliably distinguish all individuals exposed to traumatic stress from controls. In this restricted context, changes in gene expression levels in a specific organ, we found no evidence of a stress-resilient population of animals. Every individual animal exposed to the two most intense stress protocols was distinguishable from the control animals (Fig. 2b). At the level of gene expression, at least, these results are consistent with a number of psychological studies of extreme traumatic stress exposure in humans^40–43^, which suggest that the response to traumatic stress exposure is dose dependent. In this model, no individual has an absolute resilience to stress, only relative resilience that can be overcome with a sufficiently high ‘dose’ of traumatic stress.

## Methods

### Animals

Male Sprague–Dawley rats were used in all experiments (*n* = 129). All procedures were approved by the Institutional Animal Care and Use Committee (IACUC) at Stony Brook University.

### Chronic stress models

Seven different animal treatment protocols were used. Protocols were 3 weeks in duration, unless otherwise noted. Typically, stress protocols were started on postnatal day (PND) 28 and continued for three weeks (PND 28 through 49). Animals were euthanized on PND 50–51, typically 24 h following stress cessation.

*Control (C)*: Animals lived socially in groups of three per cage, with no additional stressors other than daily weighing and routine husbandry (*n* = 36).

*Social isolation (SI)*: Animals were singly housed, with no additional stressors other than daily weighing and routine husbandry (*n* = 18).

*SD*: Animals were exposed to daily sessions of defeat in the home cage of male Long Evans rats. Several variations of defeat were used including direct physical defeat, psychological threat of defeat and witnessing defeat in a conspecific. For socially housed defeat, the rats were housed socially in groups of three per cage, before and after the defeat sessions (*n* = 9).

*ID*: This protocol was identical to the SD protocol except that the rats were singly housed, before and after the defeat sessions, for the duration of the stress protocol (*n* = 8).

*GH*: Animals lived singly on metal grid of shock apparatus (Coulbourn Instruments), with no additional stressors other than daily weighing and routine husbandry (*n* = 8).

*CS*: Animals lived singly on metal grid of shock apparatus and were administered an electric foot shock of randomly varying duration and intensity, at random time intervals (*n* = 16).

*CVS*: Animals were exposed to a series of diverse physical, psychological and psychosocial stressors, including predator threat using either live animals or predator scent, water submersion, cold and warm room exposure, cage tilt and rotation, restraint, bedding disruptions, circadian rhythm disruption, shock, food and water deprivation, forced swim, isolation, and social instability (*n* = 34).

No animals were excluded from the analysis. More detailed descriptions of these protocols are given in the Supplementary Information.

### RNA isolation

Particular care was taken to minimize the effect of circadian rhythms on gene expression, which can produce false positives in RNA-sequencing results. Animal euthanasia and dissections were performed within the same 4-h time window in the middle of the diurnal light phase. Adrenal glands to be used for RNA extraction were quickly dissected, preserved in 750 µl RNALater (Life Technologies), and stored at −20 °C until homogenization.

Tissue homogenization was performed in RLT buffer (Qiagen). Total RNA was extracted from tissue samples using the RNeasy Miniprep Kit (Qiagen, Valencia, CA). RNA concentration was determined using a Nanodrop 2000 spectrophotometer and samples were then diluted to the same nominal concentration. A second round of absorbance measurements were performed to confirm the accuracy of this step.

### RNA sequencing

RNA samples from each control or stress exposure group (8–9 animals per group) were pooled for RNA sequencing. Poly(A)+ RNA was enriched from 1 μg total RNA using the poly(A) mRNA Magnetic Isolation Module (New England BioLabs, Ipswich, MA). Complementary DNA (cDNA) libraries were prepared using the Ultra Directional RNA Library Prep Kit (New England BioLabs) using 8–11 cycles of PCR amplification. dUTP was used in the cDNA synthesis to maintain strand specificity. Libraries were sequenced in multiplexed runs on an Illumina HiSeq sequencer, yielding 30–40 million 50-bp single reads per library. RNA sequencing was performed by the University of Cincinnati Genomics, Epigenomics and Sequencing Core. The reproducibility of the RNA-sequencing data obtained from this facility was verified by resequencing two technical replicate RNA libraries prepared independently in our laboratory.

### RNA-sequencing data analysis

Sequenced read fragments were mapped to the Ensembl Rnor_6.0 reference genome using the Rsubread package^44^ from the Bioconductor suite^45^ within R^46^. Expression count matrices were generated using featureCounts^47^. The count data were pre-filtered so that counts were ≥10 for all samples for a given transcript. Differential expression analysis was performed using DESeq2^48^. DESeq2 first estimates and corrects dispersion by modeling the dependence of dispersion on the average expression strength over all samples and then fits negative binomial generalized linear models for each gene. DESeq2 uses the Wald test for significance testing. The Wald test *p*-values were adjusted for multiple testing using the procedure of Benjamini and Hochberg^49^.

Because different analysis pipelines can produce discordant measures of gene expression for a subset of genes^50^, a different spliced aligner^51^ and feature identification program^52^ were used to confirm the robustness and program independence of the data analysis. The different analysis pipelines generally gave consistent results with a small subset of coding genes (<1.5%) being discordant. GO analysis was performed using the goseq package^53^ in R with FDR = 0.05. Raw and processed RNA-sequencing data have been deposited in NCBI's Gene Expression Omnibus database (GSE100454).

### Quantitative PCR

cDNA was synthesized using Superscript III Reverse Transcriptase (Life Technologies) according to the manufacturer’s instructions. Briefly, 5 μg RNA were annealed with random hexamers (Life Technologies), the mixture was rapidly renatured and reverse transcription was performed in a 20 μl reaction at 50 °C for 1 h. After enzyme inactivation for 10 min at 85 °C, the cDNA was diluted 1:16 and 4 μl of each sample were analyzed in real-time PCR reactions using a StepOne Plus instrument (Life Technologies) and the QuantiFast SYBR Green PCR kit (Qiagen). Primers were designed using the Primer3 software (MIT: http://primer3.ut.ee). The amplification products from all primer pairs (Supplementary Table S6) were validated by electrophoresis analysis and sequencing of the amplicons to confirm gene specificity. For each gene, multiple primer pairs were prescreened and only those that had high amplification efficiency and produced similar expression values were used in the experiments. Expression values were determined using a threshold crossing algorithm^54^ implemented with a custom program (http://you.stonybrook.edu/mckinnonrosati/open-source/). Expression values for each sample were normalized to the average of the 18S and 28S rRNA expression values for that sample to take account of any variations in RNA/cDNA abundance between samples.

### Behavioral tests

The ASR and EPM were performed using essentially standard procedures^55–58^. See Supplementary Information for details.

### Statistics

For comparison of multiple means, ANOVA with post-hoc *t*-tests using Benjamini–Hochberg correction^49^ were performed using R^46^. PCA was also performed using R. Effect sizes are reported as Cohen’s *d*, calculated using $D_{pooled} = \sqrt {\left( {\left( {SD_1^2 + SD_2^2} \right)/2} \right)}$.

## Electronic supplementary material

Supplemental Material

Related Manuscript File
