# Supplementary material for: Common and differential transcriptional responses to different models of traumatic stress exposure in rats
Source: Transl Psychiatry. 2018 Aug 23;8:165. doi: 10.1038/s41398-018-0223-6 (PMC6107654; doi:10.1038/s41398-018-0223-6)
Supplement: Supplementary file 1 — Supplemental Material [file 41398_2018_223_MOESM1_ESM.pdf]

Supplementary Information

Supplementary Materials and Methods

Animals

Male Sprague-Dawley rats were progeny of E13 timed-pregnant dams obtained from Envigo (formerly Harlan, Franklin Township, NJ). All animals were weaned on postnatal day (PND) 21 and litter-mates were housed two to four rats per cage, until the beginning of the experiment. Animals were distributed into experimental groups, such that body weight was evenly distributed and animals from an individual litter were assigned evenly across groups. All husbandry procedures were performed by laboratory staff. Rats were weighed at least twice a week until the week prior to the start of the experiment, when they were handled and weighed daily throughout the procedure. Identification marks were placed on the rat tails (base of the tail) with permanent markers and refreshed when needed. Animals were housed in temperature and humidity controlled rooms (lights on 0700 – 1900 h) with food and water *ad libitum*, except when the stress procedure required food/water deprivation or light cycle changes.

All procedures were performed in accordance with protocols approved by the Institutional Animal Care and Use Committee (IACUC) at Stony Brook University.

Chronic Stress Models

The six different stress models can be broadly classified (1) as either *chronic continuous stress* (SI, GH, CS), where the same stressor is present continuously, or *chronic intermittent stress* (SD, ID, CVS), where exposure to the stressor (same or variable) is interspersed with periods of relative calm. Both the ID and CVS protocols did, however, have some continuous stress elements. In the ID protocol the animals were singly housed between social defeat stress exposures. In the CVS protocol the animals were housed under conditions of social instability.

For the chronic continuous stress protocols, in particular, there is the potential for habituation (2). Nothing was done to deal with this possibility in the case of the SI and GH protocols. For the CS protocol the time between shocks and the duration of the shock were randomized and the shock intensity was changed randomly every 24 hours. It is thought that foot shock is relatively resistant to habituation compared to other stressors (3). Habituation would have been less likely in the chronic intermittent stress models. Nonetheless, for the two social defeat models, efforts were made to minimize habituation by varying the nature of the defeat protocol used. For the CVS model the wide variety of stressors used should have minimized habituation.

Typically, stress protocols were started on PND 28 and continued daily for three weeks (PND 28 through 49). Animals were euthanized on PND 50-51. Two variations on this protocol

were used for the chronic variable stress (CVS) protocol only: the stress period was one week in duration and it started on either PND 28 or PND 42.

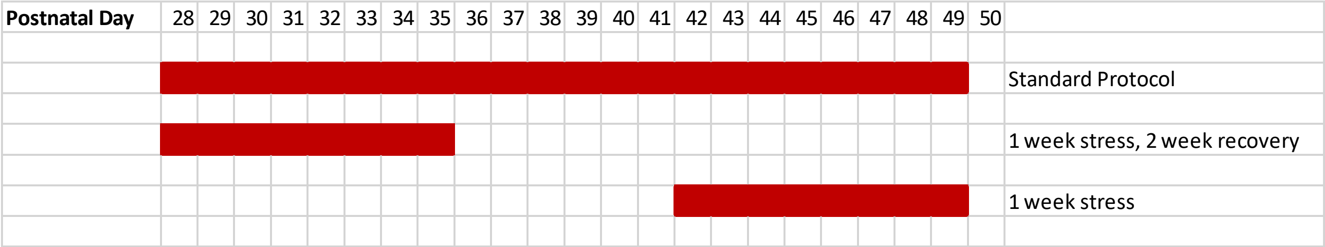

Defeat

The model consisted of a basic resident/intruder paradigm where the intruder (stressed rat) is defeated and then protected from further attacks and injury by means of a barrier (4, 5). Negative effects resulting from stress exposure can be alleviated by social housing, particularly for social defeat (6-9). In order to examine the effect of social isolation, two social defeat groups were established.

1. Social Defeat (SD) - Animals were housed socially, three per cage with the same cage mates throughout the stress protocol.
2. Isolation Defeat (ID) - Animals were housed in isolation, one animal per cage throughout the stress protocol.

Male Long Evans (LE) rats (Envigo) were used as residents. The animals were approximately 3 months old and 350 g at the time of use in the defeat protocol. During the defeat period of each session, stressed animals were placed directly in the home cage of the LE rats. Free interactions were allowed to occur until one of two criteria was met: (1) there were three separate defeats, during which the Sprague-Dawley rat was attacked and dominated by the resident rat, and then adapted a submissive posture or, (2) 10 min had elapsed.

Being placed behind a barrier in the presence of the residents is known to be adversely stressful (10), and some sessions did not involve a defeat but simply placement behind the barrier within the LE rat's cage, in the presence of the LE rat. Additionally, it has been shown that witnessing social defeat in other animals is also stressful (11, 12). Therefore, a protocol variation where rats maintained behind the barrier would also witness other stressed subjects undergoing defeat was also included.

Intruder and resident pairings were randomly assigned each day to prevent social stabilization and habituation, and to control for individual variance in defeat intensity. All rats were transferred to a test room where defeats occurred, hence outside of the home colony. Comparisons of defeat intensity (a measure of latency and frequency of attacks) using video analysis demonstrated that defeat experiences were similar among all animals in these conditions.

Several variations of the defeat test were implemented, with sporadic days of rest in order to increase unpredictability and decrease the risk of habituation.

Variations on Social Defeat Test Protocol

1. *Defeat*: The intruder was placed directly in the cage of the resident, allowing physical confrontation. After defeat, the intruder was immediately taken out of the resident's cage and returned to its home cage.

2. *Defeat then barrier:* The intruder was placed directly in the cage of the resident, allowing physical confrontation. After defeat, the intruder was placed within the barrier for 15 min. After 15 min, the intruder was returned to its home cage.

3. *Barrier then defeat*: The intruder was placed directly within the barrier in the cage of the resident for 15 min. Then the intruder was taken out from behind the barrier and placed directly with the same resident, allowing physical confrontation. After defeat, the intruder was immediately taken out of the resident's cage and returned to its home cage.

4. *Barrier only*: The intruder was placed directly within the barrier in the cage of the resident for 30 min, allowing only psychological stress. After 30 min, the intruder was returned to its home cage.

5. *Witness or defeat*: One intruder was placed directly in the cage of the resident, allowing physical confrontation. Another intruder was simultaneously placed within the barrier of the cage of the resident for the duration of the session, therefore "witnessing" the defeat but having no physical stress exposure. After defeat, both intruders were returned to their home cages.

6. *Witness or defeat then Switch*: One intruder was placed directly in the cage of the resident, allowing physical confrontation. Another intruder was simultaneously placed directly within the barrier, therefore "witnessing" the defeat. After defeat, the places of the two intruder rats were switched, such that the rat in the barrier was now directly in contact with the resident, and the rat that was previously defeated was placed within the barrier. After both defeats, both intruders were returned to their home cages.

Chronic social defeat timetable

Day	Chronic Social Defeat Procedure
1	Defeat then barrier
2	Defeat then barrier
3	Defeat
4	Witness or defeat
5	Witness or defeat

6	Barrier only
7	Barrier then defeat
8	Defeat
9	Day off
10	Day off
11	Witness or defeat then Switch
12	Witness or defeat, dark cycle
13	Witness or defeat, dark cycle
14	Day off
15	Defeat then barrier
16	Defeat, dark cycle
17	Day off
18	Defeat then barrier
19	Barrier only
20	Witness or defeat then Switch, dark cycle
21	Witness or defeat then Switch, dark cycle
22	Defeat then barrier

All interactions occurred during the animal's light cycle, at different times of the day, unless denoted.

Chronic Shock

Animals in the chronic shock (CS) group underwent an electric foot shock (EFS) procedure. Rats were housed 24 hours a day/7 days a week in isolation for three weeks in shuttle boxes (13-inch high aerated cages with a 200sq inch metal bar floor, made of Plexiglas (sides) and aluminum (base, cover)) (Coulbourn Instruments). Each shuttle box housed two rats, separately. The backs of the shuttle boxes were blacked out so rats could not see each other through the devices. A Precision Animal Shocker (Coulbourn Instruments) was used to generate the shocks. A programmable microcontroller (Arduino Mega board) was used to trigger the shock and control relays determining the path of the shock to one of eight different cages. This microcontroller also controlled the timing and duration of the shocks. The intensity (0 – 3 mA), duration (1 – 5 s), and delay between shocks was randomized, in order to increase unpredictability and minimize adaptation and habituation. Rats experienced an average of 4 and 1 shocks per hour during the dark and light cycle, respectively. The intensity (mA) of the shock was modified by the experimenter every 24 hours. The rats in this group were housed in a different room from the animals in the other experimental groups.

Grid Housing

For the grid housing (GH) protocol, animals lived in isolation for three weeks, in similar shuttle boxes to those described above for the shock animals. These rats received no shocks and were housed in a different room from the chronic shock rats.

Chronic Variable Stress

For the chronic variable stress (CVS) protocol, animals underwent exposure to different stressors for three weeks. As noted above, two groups were exposed to stress over a one-week period. The stressors used in this abbreviated protocol were identical to those used in the longer protocol.

Animals were housed in standard cages in groups of 2 or 3, unless the protocol called for housing changes. One to three stressors were administered daily either simultaneously or at varying times throughout the day. Stressors were presented randomly across the light/dark cycle to maximize unpredictability. Stressors were delivered away from the home colony. The paradigm consisted of several physical or psychological and social stressors, described below.

Physical Stressors

1. *Water submersion*: Individual rats were placed in a bucket (diameter 11.25 in, 14.5 in tall), filled 3 quarters of the way with water at 21-22°C. Rats were allowed to swim for 40 seconds, and then were gently submerged under the surface for 20 seconds (13). The animals were then removed from the water and briefly dried with a towel. Two variations of water submersion were included: (1) Rats were just barely submerged under the water and could freely move under the surface and (2) Rats were completely submerged to the bottom of the bucket with restricted ability to move.

2. *Forced swimming*: The animals were placed in a bucket (diameter 11.25 in, 14.5 in tall), filled 3 quarters of the way with water that was either cold (16-18°C), at room temperature (21-22°C), or warm (30-32°C). Animals were then forced to swim for either 3, 5, 10, or 15 min. After swimming, the animals were briefly dried with a towel.

3. *Restraint*: Rats were placed in a flexible rodent restrainer (Decapicone, Braintree Scientific, Braintree, MA) for either 30 min or 1 hr. The restrainer was secured with tape around the base of the tail, leaving enough room for the animal to defecate but also limiting movement.

4. *Cold exposure*: The animals were transferred in isolation to covered up small rodent cages (7.5 x 11.5 x 5 in) with no bedding and then to a 4°C chamber for periods of 3 or 5 hr.

5. *Warm exposure*: The animals were transferred in isolation to covered up small rodent cages (7.5 x 11.5 x 5 in) with no bedding and then to a 37°C chamber for periods of 30 min or 1 hr.

6. *Electric foot shock*: Rats were transferred individually to shock boxes similar to those described above. Within a 15 min period, animals were exposed to either 3 or 5 shocks of 1.5 mA intensity and 2 s duration.

7. *Small cage exposure*: The animals were transferred to small rodent cages (7.5 x 11.5 x 5 in) either with or without bedding, in groups of three or in isolation.

8. *Food deprivation and water deprivation*: The rats were subjected to14-16 hours of food and/or water deprivation. An empty water bottle was returned to the animal's cage as an additional stressor so the rat could use the sipper but no water would come out. The bottle would be then refilled with water after 1 hour of empty bottle exposure.

9. *Circadian rhythm disruption*: The lights were left on during the dark phase or the lights were turned off during the light phase. This was done no closer than 6 days before euthanasia to minimize any effect on clock gene expression.

10. *Cage rotation*: Cages were placed on an orbital rotator at 100rpm for 30 min or 1 hr.

11. *Cage tilt*: Home cages with or without bedding were tilted at a 45° angle by their length, for 1 up to 16 hrs.

12. *Wet bedding*: The bedding of the home cage was soaked with 500 mL of water, and the animals were exposed to wet bedding for 15 min up to 18 hrs.

13. *No bedding*: The bedding was taken out of the home cages for 15 min up to 18 hrs.

Psychological and Social Stressors

1. *Predator exposure*: Rats were exposed to free moving, live dogs (female mongrels) or snakes (python) within the predator's home environment for 30 min to 1 hour. Rats were protected within a custom aerated Plexiglas enclosure. This container prevented direct physical contact with the predator, but allowed perception of visual, auditory, and olfactory cues associated with the predator.

2. *Predator odor exposure*: Rats were exposed to a fragments of freshly shed snake skin, fresh dog feces, bobcat urine, or ferret dander odor. For bobcat urine, filter paper was soaked with the urine, and for ferret dander odor, rags that were previously housed with ferrets were used. The materials were placed directly in clean cages and presented to the rats while they were in isolation.

3. *Isolation*: The animals were individually transferred into cages similar to their home cages or to smaller rodent cages (7.5 x 11.5 x 5 in) for up to 18 hrs.

4: *Crowding*: Up to 9 animals were placed in one cage, similar to the home cage, with or without bedding, with or without access to food and water for up to 2 hrs.

5. *Social Instability*: Rats were exposed to unstable housing by changing cage mates every day or every few days, in order to reduce social support.

6. *Foreign bedding*: Rats were placed in soiled home cages previously occupied by other rats for up to 18 hrs. Alternatively, soiled bedding was placed directly within the rats' own home cages.

Chronic variable stress timetable

Day	Chronic Variable Stress Procedure
1	Cold room (3 hrs); Water submersion
2	Social isolation in small cage overnight
3	Snake exposure (1 hr); Three 2s 1.5mA shocks
4	Restraint (1 hr), Cage tilt with 3-4 rats per cage & no bedding (2 hrs)
5	Ferret dander & bobcat urine odor while in isolation in warm room (1 hr)
6	Forced swim (16°C; 3 min); Food deprivation and lights on overnight
7	Cage rotation at 100 rpm (30 min); Wet bedding overnight
8	Cold room (4 hrs); Water submersion
9	Cage tilt (8 hrs); Dog exposure (30 min)
10	Social isolation in small cage with no food and no water overnight
11	Crowding (2 hr); Lights off during day (6 hr)
12	Forced swim (21°C; 10 min); Cage rotation at 100 rpm (45 min)
13	Foreign bedding and water deprivation overnight
14	Empty bottle exposure (1 hr)
15	Ferret dander & bobcat urine odor (45 min); No bedding overnight
16	Cage tilt with lights on overnight
17	Warm room (1 hr); Lights off during day while in isolation (6 hr)
18	Forced swim (32°C; 15 min); Food deprivation overnight
19	Snake exposure (1 hr); Wet bedding overnight while in isolation
20	Cold room (3 hrs); Restraint (1 hr); Dog feces exposure (1 hr)
21	Water submersion (two times back to back); Five 2s 1.5mA shocks
22	Cage rotation at 100rpm (1 hr); Forced swim (16°C; 5 min)

Behavioral Tests

Elevated Plus Maze

The test apparatus consisted of a black plus-shaped maze with 2 closed and 2 open arms (10 x 50 cm), located 74 cm above the ground. The closed arms were 40 cm high, and the open arms had a 2 cm high rim which encouraged entry into the open arm and reduced the chance of

the rats falling off the apparatus. Rats were placed in the center of the maze, facing a closed arm, and left to explore for 5 min. Testing took place during the animal's dark cycle in dim lighting (1 lux). Coding of each video-recording of the experiment through recorded videos was done manually using BORIS (14) with blinded scorers. The same data was also analyzed using a custom program that automatically scored rat movement in the maze and calculated times in the different compartments independent of any investigator intervention (http://you.stonybrook.edu/mckinnonrosati/open-source/). The two analysis paradigms gave similar results for shared measurements.

Acoustic Startle Response

The acoustic startle response (ASR) was tested using custom hardware and software (Jacobson et al., in preparation). Briefly, the hardware was based around a high performance low-cost microcontroller (32 bit 72 MHz ARM Cortex-M4 processor) to control the experiment and record the data. A USB-based microcontroller development board with a custom circuit board was used. Software for experiment control and data analysis was implemented using Python and C++ (http://you.stonybrook.edu/mckinnonrosati/open-source/). Rats were tested in a (23 x 12 cm) plastic animal enclosure container within a (34 x 27 cm) dark chamber. Startle response was detected by the displacement of an accelerometer (ADXL335) sensor placed below the animal's enclosure. Background noise (55 dB) and startle stimuli were delivered through a speaker located 18 cm above the holding container.

Rats were first placed in the testing chamber for a 5-minute acclimation period. Rats were exposed to twenty-four 50 ms duration stimuli (6 each at 85, 95, 105, 115 dB), with an inter-stimulus interval (ISI) that varied pseudo-randomly from 15 to 40 seconds. Startle amplitude was defined as the amplitude of the peak response (greatest positive or negative deflection) following the auditory stimulus.

After each animal's trial, the chamber was cleaned with a 70% ethanol solution. Testing was conducted during the animal's light cycle.

Supplementary References

1. Marti O, Armario A (1998): Anterior pituitary response to stress: time-related changes and adaptation. *Int J Dev Neurosci*. 16:241-260.
2. Dickens MJ, Romero LM (2013): A consensus endocrine profile for chronically stressed wild animals does not exist. *Gen Comp Endocrinol*. 191:177-189.
3. Bali A, Jaggi AS (2015): Electric foot shock stress adaptation: Does it exist or not? *Life Sci*. 130:97-102.
4. Miczek KA (1979): A new test for aggression in rats without aversive stimulation: differential effects of d-amphetamine and cocaine. *Psychopharmacology (Berl)*. 60:253-259.
5. Koolhaas JM, Coppens CM, de Boer SF, Buwalda B, Meerlo P, Timmermans PJ (2013): The resident-intruder paradigm: a standardized test for aggression, violence and social stress. *J Vis Exp*.e4367.

6. de Jong JG, van der Vegt BJ, Buwalda B, Koolhaas JM (2005): Social environment determines the long-term effects of social defeat. *Physiol Behav*. 84:87-95.
7. Haller J, Leveleki C, Baranyi J, Mikics E, Bakos N (2003): Stress, social avoidance and anxiolytics: a potential model of stress-induced anxiety. *Behav Pharmacol*. 14:439-446.
8. Nakayasu T, Ishii K (2008): Effects of pair-housing after social defeat experience on elevated plus-maze behavior in rats. *Behav Processes*. 78:477-480.
9. Ruis MA, te Brake JH, Buwalda B, De Boer SF, Meerlo P, Korte SM, et al. (1999): Housing familiar male wildtype rats together reduces the long-term adverse behavioural and physiological effects of social defeat. *Psychoneuroendocrinology*. 24:285-300.
10. Tornatzky W, Miczek KA (1994): Behavioral and autonomic responses to intermittent social stress: differential protection by clonidine and metoprolol. *Psychopharmacology (Berl)*. 116:346-356.
11. Patki G, Solanki N, Salim S (2014): Witnessing traumatic events causes severe behavioral impairments in rats. *Int J Neuropsychopharmacol*. 17:2017-2029.
12. Patki G, Salvi A, Liu H, Salim S (2015): Witnessing traumatic events and post-traumatic stress disorder: Insights from an animal model. *Neurosci Lett*. 600:28-32.
13. Richter-Levin G (1998): Acute and long-term behavioral correlates of underwater trauma--potential relevance to stress and post-stress syndromes. *Psychiatry Res*. 79:73-83.
14. Friard O, Gamba M (2016): BORIS: a free, versatile open-source event-logging software for video/audio coding and live observations. *Methods Ecol Evol*. 7:1325-1330.
15. Liao Y, Smyth GK, Shi W (2013): The Subread aligner: fast, accurate and scalable read mapping by seed-and-vote. *Nucleic Acids Res*. 41:e108.
16. Liao Y, Smyth GK, Shi W (2014): featureCounts: an efficient general purpose program for assigning sequence reads to genomic features. *Bioinformatics*. 30:923-930.
17. Love MI, Huber W, Anders S (2014): Moderated estimation of fold change and dispersion for RNA-seq data with DESeq2. *Genome Biol*. 15:550.
18. Eden E, Navon R, Steinfeld I, Lipson D, Yakhini Z (2009): GOrilla: a tool for discovery and visualization of enriched GO terms in ranked gene lists. *BMC Bioinformatics*. 10:48.
19. Fonseca NA, Marioni J, Brazma A (2014): RNA-Seq gene profiling--a systematic empirical comparison. *PLoS One*. 9:e107026.
20. Kim D, Pertea G, Trapnell C, Pimentel H, Kelley R, Salzberg SL (2013): TopHat2: accurate alignment of transcriptomes in the presence of insertions, deletions and gene fusions. *Genome Biol*. 14:R36.
21. Anders S, Pyl PT, Huber W (2015): HTSeq--a Python framework to work with high-throughput sequencing data. *Bioinformatics*. 31:166-169.
22. Ruijter JM, Pfaffl MW, Zhao S, Spiess AN, Boggy G, Blom J, et al. (2013): Evaluation of qPCR curve analysis methods for reliable biomarker discovery: bias, resolution, precision, and implications. *Methods*. 59:32-46.

Supplementary Tables

Table S1 Genes differentially expressed in both the CS and CVS protocols.

gene_id	gene_name	gene_biotype	baseMean	lfcMLE	log2FoldChange	padj
ENSRNOG00000015159	Slc9a3	protein_coding	413	-2.311	-1.426	2.966E-22
ENSRNOG00000005413	Creb3l1	protein_coding	715	2.330	1.333	5.135E-18
ENSRNOG00000012404	Thrsp	protein_coding	444	2.561	1.299	9.03E-17
ENSRNOG00000013552	Scd	protein_coding	1553	1.552	1.255	1.839E-25
ENSRNOG00000046707	AABR07063425.1	miRNA	576	-1.586	-1.134	4.814E-16
ENSRNOG00000004302	Pah	protein_coding	326	-1.708	-1.027	8.271E-11
ENSRNOG00000001806	Fetub	protein_coding	114	-1.968	-0.962	5.212E-09
ENSRNOG00000010079	Car3	protein_coding	1616	1.337	0.937	3.83E-10
ENSRNOG00000052070	Aldh1a3	protein_coding	2290	-1.122	-0.912	1.456E-13
ENSRNOG00000040108	Cd36	protein_coding	1577	-1.044	-0.818	1.1E-09
ENSRNOG00000005109	Rprm	protein_coding	555	-1.102	-0.801	7.81E-08
ENSRNOG00000007118	Eva1a	protein_coding	135	1.231	0.734	3.026E-05
ENSRNOG00000032895	Cyp4f4	protein_coding	314	-0.945	-0.715	1.221E-06
ENSRNOG00000056643	Cdh8	protein_coding	173	-1.113	-0.714	2.96E-05
ENSRNOG00000004018	Tdrd5	protein_coding	279	0.969	0.710	4.782E-06
ENSRNOG00000011284	Sgms2	protein_coding	238	0.945	0.681	2.238E-05
ENSRNOG00000008174	Appl2	protein_coding	2288	-0.849	-0.664	3.777E-06
ENSRNOG00000016573	Dgat2	protein_coding	1431	-0.935	-0.664	4.495E-05
ENSRNOG00000025608	Lrat	protein_coding	5857	0.970	0.660	0.0001215
ENSRNOG00000002461	Nid1	protein_coding	13272	0.720	0.651	7.165E-12
ENSRNOG00000016541	Enc1	protein_coding	2438	-0.762	-0.562	0.0008728
ENSRNOG00000029501	Hoga1	protein_coding	1512	-0.686	-0.560	6.263E-05
ENSRNOG00000017521	Gpr55	protein_coding	1146	-0.643	-0.519	0.0004953
ENSRNOG00000033119	Plcb4	protein_coding	3661	-0.542	-0.489	2.008E-06
ENSRNOG00000027491	Vldlr	protein_coding	936	-0.569	-0.483	0.0003002
ENSRNOG00000021084	AABR07006310.1	pseudogene	3361	-0.546	-0.471	0.0002444
ENSRNOG00000017188	Cyp27a1	protein_coding	4207	-0.568	-0.462	0.0031144
ENSRNOG00000013408	Npas2	protein_coding	328	-0.645	-0.455	0.0289781
ENSRNOG00000027837	Gm14569	protein_coding	1117	-0.532	-0.442	0.0033746
ENSRNOG00000019342	Sult1a1	protein_coding	1225	-0.606	-0.439	0.0333638
ENSRNOG00000023458	Dcaf12l1	protein_coding	1718	-0.496	-0.436	0.0003917
ENSRNOG00000003762	Smarca1	protein_coding	5481	-0.495	-0.427	0.0015001
ENSRNOG00000016163	Slc1a3	protein_coding	951	-0.490	-0.404	0.0150848
ENSRNOG00000019680	Mst1	protein_coding	767	-0.512	-0.398	0.0427158
ENSRNOG00000028891	Cyp2t1	protein_coding	1178	-0.502	-0.397	0.0345208
ENSRNOG00000003832	Vash2	protein_coding	2033	0.457	0.389	0.0101707
ENSRNOG00000017431	RGD1304884	protein_coding	2842	-0.472	-0.379	0.0439216
ENSRNOG00000026455	Gpd1l	protein_coding	12230	-0.410	-0.373	0.0005296

ENSRNOG00000016311	Slc6a2	protein_coding	901	-0.435	-0.363	0.0343331
ENSRNOG00000004660	Fzd6	protein_coding	1929	0.411	0.355	0.0198653
ENSRNOG00000053086	AABR07008334.1	protein_coding	9531	-0.380	-0.348	0.0013861
ENSRNOG00000009329	Nr1d1	protein_coding	4108	-0.376	-0.341	0.0033746
ENSRNOG00000000186	Tst	protein_coding	6270	-0.350	-0.310	0.0345208
ENSRNOG00000005711	Ptprd	protein_coding	3557	-0.347	-0.309	0.0326384

Data values are for exposure to the CS protocol.

Table S2 Genes differentially expressed in the CS protocol.

gene_id	gene_name	gene_biotype	baseMean	lfcMLE	log2FC	padj
ENSRNOG00000015159	Slc9a3	protein_coding	413	-2.311	-1.426	2.97E-22
ENSRNOG00000005413	Creb3l1	protein_coding	715	2.330	1.333	5.14E-18
ENSRNOG00000012404	Thrsp	protein_coding	444	2.561	1.299	9.03E-17
ENSRNOG00000013552	Scd	protein_coding	1553	1.552	1.255	1.84E-25
ENSRNOG00000046707	AABR07063425.1	miRNA	576	-1.586	-1.134	4.81E-16
ENSRNOG00000004302	Pah	protein_coding	326	-1.708	-1.027	8.27E-11
ENSRNOG00000021475	Ldah	protein_coding	2558	1.371	0.963	1.16E-10
ENSRNOG00000001806	Fetub	protein_coding	114	-1.968	-0.962	5.21E-09
ENSRNOG00000031612	Gls2	protein_coding	786	1.358	0.954	1.65E-10
ENSRNOG00000010079	Car3	protein_coding	1616	1.337	0.937	3.83E-10
ENSRNOG00000001821	Adipoq	protein_coding	415	1.305	0.935	2.08E-10
ENSRNOG00000052070	Aldh1a3	protein_coding	2290	-1.122	-0.912	1.46E-13
ENSRNOG00000008837	Ass1	protein_coding	2113	1.159	0.866	1.30E-09
ENSRNOG00000020817	Cyp2a1	protein_coding	87	-1.896	-0.860	4.69E-07
ENSRNOG00000010805	Fabp4	protein_coding	561	1.472	0.854	4.23E-07
ENSRNOG00000059589	Lmbr1	protein_coding	611	1.060	0.837	3.29E-10
ENSRNOG00000024349	Cbarp	protein_coding	639	1.187	0.830	8.34E-08
ENSRNOG00000002393	Eprs	protein_coding	9744	1.000	0.829	9.59E-12
ENSRNOG00000017250	Gmpr	protein_coding	1211	1.060	0.827	1.13E-09
ENSRNOG00000040108	Cd36	protein_coding	1577	-1.044	-0.818	1.10E-09
ENSRNOG00000008709	Arhgap32	protein_coding	2522	1.018	0.806	1.30E-09
ENSRNOG00000005109	Rprm	protein_coding	555	-1.102	-0.801	7.81E-08
ENSRNOG00000001001	Retn	protein_coding	89	1.566	0.776	1.11E-05
ENSRNOG00000005248	Slc1a4	protein_coding	274	1.922	0.758	1.08E-05
ENSRNOG00000009153	Cidec	protein_coding	192	1.370	0.743	3.32E-05
ENSRNOG00000007118	Eva1a	protein_coding	135	1.231	0.734	3.03E-05
ENSRNOG00000012559	Man1b1	protein_coding	5430	0.898	0.722	7.13E-08
ENSRNOG00000032895	Cyp4f4	protein_coding	314	-0.945	-0.715	1.22E-06
ENSRNOG00000056643	Cdh8	protein_coding	173	-1.113	-0.714	2.96E-05
ENSRNOG00000004018	Tdrd5	protein_coding	279	0.969	0.710	4.78E-06
ENSRNOG00000008353	Iqch	protein_coding	173	-1.216	-0.701	9.80E-05
ENSRNOG00000011284	Sgms2	protein_coding	238	0.945	0.681	2.24E-05
ENSRNOG00000025443	Map1lc3a	protein_coding	2657	1.068	0.669	2.08E-04
ENSRNOG00000006857	Ndnf	protein_coding	933	-0.864	-0.667	5.23E-06
ENSRNOG00000008174	Appl2	protein_coding	2288	-0.849	-0.664	3.78E-06
ENSRNOG00000016573	Dgat2	protein_coding	1431	-0.935	-0.664	4.49E-05
ENSRNOG00000028711	Dgat1	protein_coding	2926	2.023	0.661	1.16E-04
ENSRNOG00000025608	Lrat	protein_coding	5857	0.970	0.660	1.21E-04
ENSRNOG00000000661	Hps4	protein_coding	713	0.951	0.657	1.07E-04
ENSRNOG00000002461	Nid1	protein_coding	13272	0.720	0.651	7.17E-12

ENSRNOG00000011310	Pde10a	protein_coding	948	1.058	0.649	4.44E-04
ENSRNOG00000022256	Cxcl10	protein_coding	376	-0.963	-0.647	1.89E-04
ENSRNOG00000011251	Hcrtr2	protein_coding	1042	-0.825	-0.645	9.24E-06
ENSRNOG00000002218	Stbd1	protein_coding	737	0.966	0.633	4.44E-04
ENSRNOG00000019018	Plat	protein_coding	802	0.870	0.632	1.17E-04
ENSRNOG00000001052	Slc25a30	protein_coding	12286	0.868	0.609	4.46E-04
ENSRNOG00000007454	Aloxe3	protein_coding	228	1.001	0.609	1.50E-03
ENSRNOG00000047860	Plin5	protein_coding	228	-0.880	-0.606	4.98E-04
ENSRNOG00000058882	Emd	protein_coding	869	0.985	0.605	1.60E-03
ENSRNOG00000007271	Map3k9	protein_coding	664	-0.855	-0.600	4.99E-04
ENSRNOG00000055991	Tmprss11d	protein_coding	135	-1.035	-0.593	2.79E-03
ENSRNOG00000019587	Ptprn	protein_coding	1009	0.681	0.592	2.14E-07
ENSRNOG00000011778	Blvra	protein_coding	679	0.700	0.589	4.30E-06
ENSRNOG00000011475	Srcin1	protein_coding	661	-0.795	-0.584	4.52E-04
ENSRNOG00000018694	Lipg	protein_coding	346	-0.987	-0.583	3.11E-03
ENSRNOG00000001006	Nptx2	protein_coding	613	0.764	0.573	4.88E-04
ENSRNOG00000016541	Enc1	protein_coding	2438	-0.762	-0.562	8.73E-04
ENSRNOG00000029501	Hoga1	protein_coding	1512	-0.686	-0.560	6.26E-05
ENSRNOG00000018524	Ezr	protein_coding	1366	0.665	0.556	2.98E-05
ENSRNOG00000015518	Rbp4	protein_coding	121	1.361	0.543	7.93E-03
ENSRNOG00000006403	Nectin1	protein_coding	303	0.758	0.540	3.11E-03
ENSRNOG00000019978	Parg	protein_coding	3174	0.618	0.539	3.78E-06
ENSRNOG00000011585	Fat3	protein_coding	145	-1.161	-0.539	1.19E-02
ENSRNOG00000014163	Zfp536	protein_coding	245	0.959	0.526	1.54E-02
ENSRNOG00000021441	Reln	protein_coding	6055	0.652	0.526	4.44E-04
ENSRNOG00000015717	Ptpre	protein_coding	1429	-0.599	-0.524	6.41E-06
ENSRNOG00000017521	Gpr55	protein_coding	1146	-0.643	-0.519	4.95E-04
ENSRNOG00000029005	Rn50_X_0554.1	processed_pseudogene	344	0.893	0.514	1.93E-02
ENSRNOG00000052247	Manba	protein_coding	1032	-0.599	-0.512	5.36E-05
ENSRNOG00000033564	Cfd	protein_coding	252	0.792	0.511	1.40E-02
ENSRNOG00000016483	Myo16	protein_coding	296	0.747	0.505	1.21E-02
ENSRNOG00000019422	Egr1	protein_coding	350	0.830	0.504	2.09E-02
ENSRNOG00000000981	Scarb1	protein_coding	85933	0.579	0.503	3.40E-05
ENSRNOG00000007286	Mdm1	protein_coding	979	-0.600	-0.502	2.76E-04
ENSRNOG00000015086	Plin1	protein_coding	196	0.913	0.500	2.80E-02
ENSRNOG00000000503	Ppard	protein_coding	2495	0.630	0.499	1.88E-03
ENSRNOG00000001091	Hip1r	protein_coding	2754	0.560	0.493	2.37E-05
ENSRNOG00000047476	Wasf1	protein_coding	603	0.660	0.491	6.97E-03
ENSRNOG00000022239	Tusc5	protein_coding	150	0.902	0.490	3.35E-02
ENSRNOG00000033119	Plcb4	protein_coding	3661	-0.542	-0.489	2.01E-06
ENSRNOG00000011984	Cxcl14	protein_coding	1579	-0.558	-0.486	5.42E-05
ENSRNOG00000009068	Phlda3	protein_coding	718	0.590	0.485	1.11E-03
ENSRNOG00000027491	Vldlr	protein_coding	936	-0.569	-0.483	3.00E-04

ENSRNOG00000011411	Adgrg6	protein_coding	308	-0.638	-0.483	6.57E-03
ENSRNOG00000011858	Unc5d	protein_coding	265	-0.690	-0.482	1.58E-02
ENSRNOG00000038445	Rnf225	protein_coding	83	1.256	0.481	3.12E-02
ENSRNOG00000009734	Akr1b8	protein_coding	35317	0.526	0.478	1.34E-06
ENSRNOG00000055567	Fmnl2	protein_coding	2260	0.592	0.474	3.16E-03
ENSRNOG00000037688	Ak9	protein_coding	117	-0.863	-0.474	4.39E-02
ENSRNOG00000049075	Fabp5	protein_coding	162	0.949	0.472	4.79E-02
ENSRNOG00000021084	AABR07006310.1	pseudogene	3361	-0.546	-0.471	2.44E-04
ENSRNOG00000011253	RGD1304810	protein_coding	519	-0.821	-0.471	4.39E-02
ENSRNOG00000032922	Dclk1	protein_coding	578	0.634	0.469	1.41E-02
ENSRNOG00000015354	Aox1	protein_coding	2266	-0.560	-0.465	1.54E-03
ENSRNOG00000005332	Csdc2	protein_coding	1759	-0.580	-0.465	3.87E-03
ENSRNOG00000004448	Acss3	protein_coding	497	-0.597	-0.463	7.93E-03
ENSRNOG00000017188	Cyp27a1	protein_coding	4207	-0.568	-0.462	3.11E-03
ENSRNOG00000017808	Klf15	protein_coding	621	-0.563	-0.456	3.88E-03
ENSRNOG00000013408	Npas2	protein_coding	328	-0.645	-0.455	2.90E-02
ENSRNOG00000032768	Stxbp4	protein_coding	368	-0.601	-0.453	1.54E-02
ENSRNOG00000023548	Sned1	protein_coding	519	-0.631	-0.452	2.77E-02
ENSRNOG00000003875	Ocrl	protein_coding	2575	-0.548	-0.451	3.37E-03
ENSRNOG00000028017	Tmem109	protein_coding	4227	0.495	0.450	1.03E-05
ENSRNOG00000028611	AABR07037203.1	processed_pseudogene	502	0.656	0.446	4.39E-02
ENSRNOG00000014367	Ephb6	protein_coding	3741	-0.540	-0.446	3.47E-03
ENSRNOG00000010816	Kcnk15	protein_coding	665	0.543	0.445	4.45E-03
ENSRNOG00000033740	Lurap1l	protein_coding	455	0.607	0.443	3.05E-02
ENSRNOG00000027837	Gm14569	protein_coding	1117	-0.532	-0.442	3.37E-03
ENSRNOG00000006076	Steap2	protein_coding	699	-0.571	-0.439	1.79E-02
ENSRNOG00000019342	Sult1a1	protein_coding	1225	-0.606	-0.439	3.34E-02
ENSRNOG00000005371	Klhl29	protein_coding	1543	0.604	0.438	3.45E-02
ENSRNOG00000023458	Dcaf12l1	protein_coding	1718	-0.496	-0.436	3.92E-04
ENSRNOG00000014117	Hmox1	protein_coding	664	-0.520	-0.431	4.58E-03
ENSRNOG00000018242	Camkk1	protein_coding	980	-0.587	-0.431	3.45E-02
ENSRNOG00000000796	Ranbp2	protein_coding	9548	0.532	0.429	1.10E-02
ENSRNOG00000003762	Smarca1	protein_coding	5481	-0.495	-0.427	1.50E-03
ENSRNOG00000052745	Met	protein_coding	3356	-0.502	-0.425	3.37E-03
ENSRNOG00000014191	Zfp395	protein_coding	2593	0.502	0.425	3.62E-03
ENSRNOG00000011696	Lifr	protein_coding	1311	-0.499	-0.425	2.97E-03
ENSRNOG00000048495	Rrp12	protein_coding	1322	0.503	0.424	4.29E-03
ENSRNOG00000013928	Dsp	protein_coding	10340	0.513	0.422	9.44E-03
ENSRNOG00000016576	Lrrc16a	protein_coding	2710	-0.520	-0.419	1.45E-02
ENSRNOG00000037352	RGD1562747	protein_coding	1463	1.584	0.417	4.39E-02
ENSRNOG00000015562	Cdh17	protein_coding	429	-0.533	-0.416	2.74E-02
ENSRNOG00000018019	Hspa12a	protein_coding	425	0.516	0.413	2.12E-02
ENSRNOG00000007290	Atp1a2	protein_coding	663	0.495	0.412	9.44E-03

ENSRNOG00000013712	Tex261	protein_coding	1662	0.518	0.412	2.47E-02
ENSRNOG00000021027	Dbp	protein_coding	11340	-0.473	-0.411	2.42E-03
ENSRNOG00000017311	Me3	protein_coding	953	-0.482	-0.410	4.58E-03
ENSRNOG00000023360	Fus	protein_coding	2882	0.482	0.408	6.79E-03
ENSRNOG00000009431	Tbc1d4	protein_coding	776	0.521	0.404	3.95E-02
ENSRNOG00000016163	Slc1a3	protein_coding	951	-0.490	-0.404	1.51E-02
ENSRNOG00000012333	Kbtbd11	protein_coding	483	0.525	0.402	4.55E-02
ENSRNOG00000016825	Cd3eap	protein_coding	355	0.517	0.402	4.00E-02
ENSRNOG00000007545	Angptl4	protein_coding	1176	0.514	0.401	3.95E-02
ENSRNOG00000013663	Tmem86a	protein_coding	7644	-0.501	-0.401	2.80E-02
ENSRNOG00000019996	Slc16a1	protein_coding	1295	0.462	0.399	3.94E-03
ENSRNOG00000019680	Mst1	protein_coding	767	-0.512	-0.398	4.27E-02
ENSRNOG00000028891	Cyp2t1	protein_coding	1178	-0.502	-0.397	3.45E-02
ENSRNOG00000010558	Ppif	protein_coding	1176	0.485	0.396	2.32E-02
ENSRNOG00000013179	Tinagl1	protein_coding	4564	0.464	0.394	9.63E-03
ENSRNOG00000008845	Pdrg1	protein_coding	1678	0.450	0.392	3.70E-03
ENSRNOG00000005362	Rab3ip	protein_coding	696	-0.483	-0.391	3.04E-02
ENSRNOG00000006997	App	protein_coding	23130	0.435	0.390	8.73E-04
ENSRNOG00000003832	Vash2	protein_coding	2033	0.457	0.389	1.02E-02
ENSRNOG00000033517	LOC100360791	processed_pseudogene	4800	0.453	0.388	7.93E-03
ENSRNOG00000055391	Eif4ebp2	protein_coding	1221	-0.476	-0.387	3.05E-02
ENSRNOG00000017765	Net1	protein_coding	8420	0.429	0.381	2.87E-03
ENSRNOG00000013911	Nagk	protein_coding	1364	0.443	0.380	1.10E-02
ENSRNOG00000017431	RGD1304884	protein_coding	2842	-0.472	-0.379	4.39E-02
ENSRNOG00000003546	Tnfrsf12a	protein_coding	1454	0.452	0.376	2.88E-02
ENSRNOG00000014648	Efnb2	protein_coding	1642	0.462	0.374	4.59E-02
ENSRNOG00000026455	Gpd1l	protein_coding	12230	-0.410	-0.373	5.30E-04
ENSRNOG00000013269	Tnfsf10	protein_coding	6337	-0.421	-0.373	3.52E-03
ENSRNOG00000057125	Ddr1	protein_coding	3155	-0.450	-0.370	3.84E-02
ENSRNOG00000021931	Uba6	protein_coding	1902	-0.436	-0.370	2.09E-02
ENSRNOG00000017911	Tcaf1	protein_coding	1858	0.417	0.369	3.98E-03
ENSRNOG00000005391	Prex2	protein_coding	1196	-0.435	-0.367	2.49E-02
ENSRNOG00000045636	Fasn	protein_coding	5215	0.425	0.365	1.67E-02
ENSRNOG00000015020	Idh1	protein_coding	10731	-0.423	-0.364	1.58E-02
ENSRNOG00000021106	Gramd1a	protein_coding	3546	-0.412	-0.364	5.77E-03
ENSRNOG00000016311	Slc6a2	protein_coding	901	-0.435	-0.363	3.43E-02
ENSRNOG00000013027	Rgl3	protein_coding	2822	0.438	0.362	4.39E-02
ENSRNOG00000025815	Cdr2l	protein_coding	2036	0.435	0.360	4.39E-02
ENSRNOG00000001203	Rrp1	protein_coding	1983	0.419	0.357	2.77E-02
ENSRNOG00000021269	Chgb	protein_coding	35771	0.397	0.356	3.87E-03
ENSRNOG00000004660	Fzd6	protein_coding	1929	0.411	0.355	1.99E-02
ENSRNOG00000007393	Ndrg1	protein_coding	1003	0.424	0.354	4.45E-02
ENSRNOG00000001113	Mmd2	protein_coding	12921	-0.383	-0.353	4.98E-04

ENSRNOG00000022922	Slc25a12	protein_coding	4399	0.389	0.352	2.87E-03
ENSRNOG00000053086	AABR07008334.1	protein_coding	9531	-0.380	-0.348	1.39E-03
ENSRNOG00000008543	Pdlim2	protein_coding	2698	-0.404	-0.348	2.82E-02
ENSRNOG00000006931	Eepd1	protein_coding	39918	-0.391	-0.346	1.08E-02
ENSRNOG00000003259	C1qtnf1	protein_coding	10172	-0.381	-0.345	3.51E-03
ENSRNOG00000003468	Capn8	protein_coding	693	-0.408	-0.344	4.65E-02
ENSRNOG00000024886	Ext1	protein_coding	844	0.404	0.343	4.41E-02
ENSRNOG00000009329	Nr1d1	protein_coding	4108	-0.376	-0.341	3.37E-03
ENSRNOG00000021637	Ddx46	protein_coding	5235	0.389	0.340	2.43E-02
ENSRNOG00000023257	Adamts9	protein_coding	2911	0.392	0.338	3.55E-02
ENSRNOG00000056457	Gpd1	protein_coding	12819	-0.367	-0.337	1.62E-03
ENSRNOG00000010633	Acsl1	protein_coding	4897	0.389	0.334	4.22E-02
ENSRNOG00000005513	Srsf5	protein_coding	6095	0.388	0.333	4.39E-02
ENSRNOG00000002708	Phf8	protein_coding	1621	-0.375	-0.331	2.11E-02
ENSRNOG00000014293	Nkd1	protein_coding	1172	-0.379	-0.329	3.56E-02
ENSRNOG00000000583	Cdk19	protein_coding	2330	-0.379	-0.329	3.45E-02
ENSRNOG00000017105	Dpyd	protein_coding	5750	-0.362	-0.327	8.49E-03
ENSRNOG00000021773	Bop1	protein_coding	2348	0.367	0.323	3.05E-02
ENSRNOG00000031662	Slc6a5	protein_coding	27121	-0.348	-0.321	3.11E-03
ENSRNOG00000013742	Large	protein_coding	3144	-0.354	-0.312	3.66E-02
ENSRNOG00000000186	Tst	protein_coding	6270	-0.350	-0.310	3.45E-02
ENSRNOG00000005711	Ptprd	protein_coding	3557	-0.347	-0.309	3.26E-02
ENSRNOG00000002292	Hnrnpd	protein_coding	3014	0.345	0.307	3.35E-02
ENSRNOG00000015654	Ghr	protein_coding	4315	-0.337	-0.302	3.05E-02
ENSRNOG00000031743	Gbp2	protein_coding	3272	-0.328	-0.293	4.55E-02
ENSRNOG00000007110	Ankrd6	protein_coding	2836	-0.322	-0.291	3.23E-02
ENSRNOG00000010557	Smarcd2	protein_coding	5729	-0.318	-0.288	3.05E-02

Table S3 Genes differentially expressed in the CVS protocol.

gene_id	gene_name	gene_biotype	baseMean	lfcMLE	log2FoldChange	padj
ENSRNOG00000015159	Slc9a3	protein_coding	374	-3.209	-1.646	1.74E-40
ENSRNOG00000046707	AABR07063425.1	miRNA	514	-2.240	-1.243	1.40E-22
ENSRNOG00000004302	Pah	protein_coding	301	-2.016	-1.062	6.86E-16
ENSRNOG00000040108	Cd36	protein_coding	1416	-1.437	-1.026	2.07E-18
ENSRNOG00000012404	Thrsp	protein_coding	289	1.831	0.797	2.02E-08
ENSRNOG00000056643	Cdh8	protein_coding	155	-1.552	-0.794	2.77E-08
ENSRNOG00000013552	Scd	protein_coding	1325	1.305	0.772	4.54E-08
ENSRNOG00000005109	Rprm	protein_coding	521	-1.195	-0.760	2.80E-08
ENSRNOG00000018824	Slc7a5	protein_coding	1581	-1.053	-0.755	2.67E-09
ENSRNOG00000001806	Fetub	protein_coding	105	-2.251	-0.752	5.82E-08
ENSRNOG00000019120	Hmgcs2	protein_coding	275	-1.320	-0.739	2.30E-07
ENSRNOG00000012847	Scgb1c1	protein_coding	437	-1.051	-0.733	2.02E-08
ENSRNOG00000006622	Cry1	protein_coding	645	1.022	0.712	6.76E-08
ENSRNOG00000013408	Npas2	protein_coding	282	-1.233	-0.698	1.73E-06
ENSRNOG00000009425	Fgf7	protein_coding	235	-1.254	-0.674	6.77E-06
ENSRNOG00000016311	Slc6a2	protein_coding	786	-0.877	-0.665	4.93E-08
ENSRNOG00000017209	Tubb3	protein_coding	1507	-0.786	-0.660	6.52E-11
ENSRNOG00000029501	Hoga1	protein_coding	1397	-0.849	-0.653	5.42E-08
ENSRNOG00000001414	Serpine1	protein_coding	321	-1.175	-0.610	1.08E-04
ENSRNOG00000007118	Eva1a	protein_coding	129	1.236	0.608	1.16E-04
ENSRNOG00000016163	Slc1a3	protein_coding	824	-0.967	-0.600	7.50E-05
ENSRNOG00000010079	Car3	protein_coding	1311	0.946	0.592	1.08E-04
ENSRNOG00000028891	Cyp2t1	protein_coding	1061	-0.777	-0.590	3.18E-06
ENSRNOG00000016541	Enc1	protein_coding	2324	-0.763	-0.578	6.84E-06
ENSRNOG00000016456	Il33	protein_coding	424	-0.805	-0.568	6.37E-05
ENSRNOG00000017283	Kcnt1	protein_coding	262	0.870	0.566	2.03E-04
ENSRNOG00000052070	Aldh1a3	protein_coding	2330	-0.764	-0.562	3.66E-05
ENSRNOG00000032895	Cyp4f4	protein_coding	304	-0.859	-0.560	2.14E-04
ENSRNOG00000019342	Sult1a1	protein_coding	1121	-0.813	-0.557	1.42E-04
ENSRNOG00000014656	Cox8b	protein_coding	709	-0.926	-0.555	5.01E-04
ENSRNOG00000020254	Per2	protein_coding	2578	0.674	0.553	9.60E-07
ENSRNOG00000001295	S100b	protein_coding	552	0.757	0.542	1.57E-04
ENSRNOG00000000024	Hebp1	protein_coding	200	0.922	0.539	1.11E-03
ENSRNOG00000003765	Nr0b1	protein_coding	696	-0.725	-0.533	1.16E-04
ENSRNOG00000016412	Fxyd6	protein_coding	3498	-0.619	-0.531	1.04E-07
ENSRNOG00000015003	Pex11a	protein_coding	423	-0.948	-0.527	1.75E-03
ENSRNOG00000002461	Nid1	protein_coding	12419	0.667	0.521	6.30E-05
ENSRNOG00000058891	Cys1	protein_coding	436	0.729	0.518	5.01E-04
ENSRNOG00000017521	Gpr55	protein_coding	1074	-0.723	-0.517	4.15E-04
ENSRNOG00000008534	Dusp15	protein_coding	409	1.029	0.515	3.04E-03

ENSRNOG00000009329	Nr1d1	protein_coding	3549	-0.847	-0.503	3.10E-03
ENSRNOG00000016573	Dgat2	protein_coding	1404	-0.786	-0.498	2.76E-03
ENSRNOG00000007544	Il23r	protein_coding	148	-0.958	-0.493	5.70E-03
ENSRNOG00000003832	Vash2	protein_coding	2030	0.592	0.486	5.29E-05
ENSRNOG00000011754	Myom2	protein_coding	1872	-0.623	-0.478	5.03E-04
ENSRNOG00000019622	Ackr3	protein_coding	184	0.882	0.478	8.73E-03
ENSRNOG00000025608	Lrat	protein_coding	5076	0.716	0.476	4.36E-03
ENSRNOG00000004018	Tdrd5	protein_coding	249	0.795	0.476	7.64E-03
ENSRNOG00000005413	Creb3l1	protein_coding	326	0.703	0.474	4.17E-03
ENSRNOG00000006709	Pzp	protein_coding	79	1.135	0.471	8.73E-03
ENSRNOG00000017672	Akr1c14	protein_coding	198	-0.861	-0.471	1.01E-02
ENSRNOG00000027837	Gm14569	protein_coding	1052	-0.593	-0.470	3.07E-04
ENSRNOG00000011284	Sgms2	protein_coding	211	0.766	0.470	8.35E-03
ENSRNOG00000000728	Clic2	protein_coding	3200	0.606	0.469	6.66E-04
ENSRNOG00000011841	Map2	protein_coding	2050	0.694	0.468	5.01E-03
ENSRNOG00000018020	Apbb1	protein_coding	1714	-0.681	-0.463	5.01E-03
ENSRNOG00000006646	Vopp1	protein_coding	1009	0.589	0.462	6.36E-04
ENSRNOG00000010438	Cpt1b	protein_coding	617	-0.767	-0.459	1.15E-02
ENSRNOG00000003869	Sod3	protein_coding	17855	0.534	0.452	6.37E-05
ENSRNOG00000003762	Smarca1	protein_coding	5138	-0.576	-0.448	1.38E-03
ENSRNOG00000020714	Trpm4	protein_coding	172	0.799	0.442	2.28E-02
ENSRNOG00000018798	Bcan	protein_coding	197	-0.774	-0.441	2.09E-02
ENSRNOG00000031207	LOC500035	protein_coding	15164	-1.446	-0.440	1.15E-02
ENSRNOG00000017628	Tagln	protein_coding	2915	-0.552	-0.439	1.08E-03
ENSRNOG00000019328	Phgdh	protein_coding	1611	-0.613	-0.436	8.11E-03
ENSRNOG00000008174	Appl2	protein_coding	2279	-0.632	-0.434	1.15E-02
ENSRNOG00000012787	Tmem164	protein_coding	5444	-0.531	-0.430	1.03E-03
ENSRNOG00000019582	Mthfd1l	protein_coding	456	0.609	0.427	1.30E-02
ENSRNOG00000059017	AC106292.2	protein_coding	2722	0.527	0.420	2.62E-03
ENSRNOG00000033057	RGD1560797	protein_coding	388	-0.601	-0.417	1.70E-02
ENSRNOG00000051405	AABR07021465.1	protein_coding	197	0.874	0.417	4.62E-02
ENSRNOG00000018405	Apoc4	protein_coding	2128	-0.510	-0.416	1.47E-03
ENSRNOG00000019491	Stard10	protein_coding	4434	-0.478	-0.415	9.06E-05
ENSRNOG00000019680	Mst1	protein_coding	725	-0.552	-0.415	8.43E-03
ENSRNOG00000007970	Plxnc1	protein_coding	284	0.852	0.414	4.94E-02
ENSRNOG00000006472	Hspa2	protein_coding	277	0.729	0.412	4.85E-02
ENSRNOG00000050485	Gas1	protein_coding	664	-0.701	-0.410	4.62E-02
ENSRNOG00000016717	Gas2	protein_coding	528	-0.665	-0.406	4.62E-02
ENSRNOG00000005998	Smoc1	protein_coding	2232	0.528	0.404	9.62E-03
ENSRNOG00000005711	Ptprd	protein_coding	3286	-0.485	-0.403	1.35E-03
ENSRNOG00000003510	Fmo2	protein_coding	1769	0.549	0.403	1.70E-02
ENSRNOG00000017188	Cyp27a1	protein_coding	4060	-0.516	-0.398	9.67E-03
ENSRNOG00000000622	Hint1	protein_coding	516	-0.624	-0.397	4.95E-02

ENSRNOG00000027491	Vldlr	protein_coding	908	-0.489	-0.395	4.55E-03
ENSRNOG00000001627	Abi3bp	protein_coding	5852	0.464	0.391	1.54E-03
ENSRNOG00000018251	Mrc1	protein_coding	1735	0.482	0.391	5.01E-03
ENSRNOG00000017431	RGD1304884	protein_coding	2693	-0.501	-0.388	1.24E-02
ENSRNOG00000004660	Fzd6	protein_coding	1875	0.468	0.385	4.36E-03
ENSRNOG00000003515	Ephx1	protein_coding	1705	0.447	0.376	3.22E-03
ENSRNOG00000010017	Wee1	protein_coding	1021	0.458	0.376	6.63E-03
ENSRNOG00000026455	Gpd1l	protein_coding	11644	-0.418	-0.374	1.16E-04
ENSRNOG00000003526	Sytl4	protein_coding	1367	0.492	0.372	3.15E-02
ENSRNOG00000026036	Pdyn	protein_coding	1274	0.497	0.371	3.90E-02
ENSRNOG00000006766	Laptm4b	protein_coding	4303	-0.491	-0.370	3.45E-02
ENSRNOG00000005929	Them6	protein_coding	691	-0.485	-0.369	3.08E-02
ENSRNOG00000059500	Cdkn1c	protein_coding	3096	0.426	0.365	2.70E-03
ENSRNOG00000005424	Odc1	protein_coding	4897	-0.446	-0.361	1.38E-02
ENSRNOG00000000186	Tst	protein_coding	5882	-0.421	-0.360	3.09E-03
ENSRNOG00000019219	Vamp1	protein_coding	1346	-0.465	-0.359	3.37E-02
ENSRNOG00000002141	Cd200	protein_coding	2981	0.429	0.352	1.44E-02
ENSRNOG00000015618	Wnt5a	protein_coding	1711	0.406	0.340	1.38E-02
ENSRNOG00000015602	Cdh2	protein_coding	1464	-0.396	-0.335	1.17E-02
ENSRNOG00000009388	Sptssb	protein_coding	50	2.049	0.334	1.87E-02
ENSRNOG00000030111	Cyp11b2	protein_coding	55958	0.411	0.333	4.01E-02
ENSRNOG00000000648	Jmjd1c	protein_coding	2707	0.387	0.330	1.38E-02
ENSRNOG00000006086	Lynx1	protein_coding	12602	-0.387	-0.329	1.44E-02
ENSRNOG00000020246	Myl9	protein_coding	1388	-0.389	-0.328	1.79E-02
ENSRNOG00000053086	AABR07008334.1	protein_coding	9118	-0.368	-0.328	2.01E-03
ENSRNOG00000010284	St3gal5	protein_coding	3517	-0.370	-0.323	6.77E-03
ENSRNOG00000000585	Amd1	protein_coding	5269	-0.369	-0.321	9.83E-03
ENSRNOG00000021084	AABR07006310.1	pseudogene	3322	-0.391	-0.320	4.62E-02
ENSRNOG00000023458	Dcaf12l1	protein_coding	1685	-0.374	-0.320	1.79E-02
ENSRNOG00000002449	Maged2	protein_coding	11102	-0.372	-0.319	1.61E-02
ENSRNOG00000021262	Slc23a2	protein_coding	19102	0.344	0.309	3.71E-03
ENSRNOG00000059660	AABR07065531.26	lincRNA	4914	0.358	0.303	4.62E-02
ENSRNOG00000014090	Retsat	protein_coding	8082	-0.345	-0.300	2.09E-02
ENSRNOG00000033119	Plcb4	protein_coding	3662	-0.335	-0.293	2.38E-02
ENSRNOG00000014718	Acsl3	protein_coding	3326	0.341	0.293	4.38E-02
ENSRNOG00000013766	Acaa2	protein_coding	18185	-0.331	-0.287	4.02E-02
ENSRNOG00000014254	Cpt1a	protein_coding	9811	-0.327	-0.283	4.74E-02
ENSRNOG00000016957	Igfbp2	protein_coding	10649	-0.303	-0.271	2.42E-02
ENSRNOG00000038746	Bco2	protein_coding	3806	0.304	0.268	4.84E-02
ENSRNOG00000026842	Nnt	protein_coding	22407	0.290	0.259	4.02E-02
ENSRNOG00000036816	Wls	protein_coding	10784	0.288	0.257	4.92E-02

Table S4 Over and Under Represented Categories: Gene Ontology Analysis of CS Protocol

category	Over represented pvalue	Under represented pvalue	Number DE in Category	Number in Category	GO term	GO ontology
GO:0044281	2.73E-10	1	40	1609	small molecule metabolic process	BP
GO:0006629	1.63E-07	0.999999957	27	1043	lipid metabolic process	BP
GO:0005737	4.07E-06	0.999998058	105	9013	cytoplasm	CC
GO:0005576	7.04E-06	0.999996857	55	3706	extracellular region	CC
GO:0005615	7.21E-06	0.999996893	49	3141	extracellular space	CC
GO:0048856	1.63E-05	0.999992208	63	4604	anatomical structure development	BP
GO:0043167	2.29E-05	0.999988836	65	4862	ion binding	MF
GO:0007155	3.36E-05	0.999989207	22	997	cell adhesion	BP
GO:0006810	3.84E-05	0.999981639	55	3934	transport	BP
GO:0005811	7.31E-05	0.99999535	5	53	lipid particle	CC
GO:0016491	9.16E-05	0.999972074	18	774	oxidoreductase activity	MF
GO:0043226	0.000110833	0.999942411	119	11339	organelle	CC
GO:0042592	0.000125322	0.999951732	26	1412	homeostatic process	BP
GO:0000902	0.000155866	0.999950325	18	807	cell morphogenesis	BP
GO:0030154	0.000195262	0.999904797	45	3171	cell differentiation	BP
GO:0008289	0.000275589	0.999922094	14	559	lipid binding	MF
GO:0005622	0.000321223	0.999830649	127	12590	intracellular	CC
GO:0006950	0.000379802	0.999812206	42	2971	response to stress	BP
GO:0008150	0.000781855	0.999727157	160	17509	biological_process	BP
GO:0005783	0.000795531	0.999684091	21	1167	endoplasmic reticulum	CC
GO:0005975	0.001236169	0.999610779	13	575	carbohydrate metabolic process	BP
GO:0040011	0.001748991	0.999243325	22	1333	locomotion	BP
GO:0007267	0.002325212	0.999034541	19	1098	cell-cell signaling	BP
GO:0008219	0.00248868	0.998833858	25	1633	cell death	BP
GO:0030198	0.002526574	0.999548035	6	165	extracellular matrix organization	BP
GO:0048870	0.003854718	0.998322506	19	1158	cell motility	BP
GO:0006520	0.00468123	0.998897022	7	246	cellular amino acid metabolic process	BP
GO:0009056	0.00617909	0.996884294	25	1752	catabolic process	BP
GO:0005575	0.011560741	0.995740383	163	18463	cellular_component	CC
GO:0005623	0.012467283	0.992815643	144	15710	cell	CC
GO:0031410	0.021699152	0.988967203	18	1281	cytoplasmic vesicle	CC

GO:0019899	0.023369604	0.987220793	22	1675	enzyme binding	MF
GO:0005768	0.024058946	0.990365357	10	579	endosome	CC
GO:0008092	0.026781078	0.988092676	12	752	cytoskeletal protein binding	MF
GO:0008283	0.029788159	0.983525881	21	1612	cell proliferation	BP
GO:0055085	0.03667965	0.980550215	17	1262	transmembrane transport	BP
GO:0040007	0.03921207	0.98155062	12	803	growth	BP
GO:0030674	0.045731213	0.99160785	3	96	protein binding, bridging	MF
GO:0006790	0.04987188	0.984885651	5	239	sulfur compound metabolic process	BP
GO:0043234	0.980506966	0.034550223	16	2918	protein complex	CC
GO:0004871	0.982174292	0.03281686	14	2643	signal transducer activity	MF
GO:0050877	0.991087272	0.019044039	10	2192	neurological system process	BP
GO:0005198	0.996203613	0.017172752	2	897	structural molecule activity	MF
GO:0051276	0.997700646	0.011074009	2	963	chromosome organization	BP
GO:0003735	1	0.012741517	0	508	structural constituent of ribosome	MF
GO:0005840	1	0.006361251	0	589	ribosome	CC

Table S5 Over and Under Represented Categories: Gene Ontology Analysis of CVS Protocol

category	Over represented pvalue	Under represented pvalue	Number DE in Category	Number in Category	GO term	GO ontology
GO:0044281	3.39E-07	0.999999918	24	1609	small molecule metabolic process	BP
GO:0016491	3.67E-06	0.999999282	15	774	oxidoreductase activity	MF
GO:0006629	7.59E-06	0.999998243	17	1043	lipid metabolic process	BP
GO:0048856	7.79E-05	0.999968078	39	4604	anatomical structure development	BP
GO:0005737	1.73E-04	0.99992317	61	9013	cytoplasm	CC
GO:0030154	2.76E-04	0.999888508	29	3171	cell differentiation	BP
GO:0019748	9.83E-04	0.999957031	3	41	secondary metabolic process	BP
GO:0006810	1.97E-03	0.999071391	31	3934	transport	BP
GO:0005575	2.47E-03	0.999744088	95	18463	cellular_component	CC
GO:0005576	3.44E-03	0.998339022	29	3706	extracellular region	CC
GO:0008283	3.96E-03	0.998446845	16	1612	cell proliferation	BP
GO:0051186	0.004300888	0.999147579	6	324	cofactor metabolic process	BP
GO:0006790	0.005518581	0.999050759	5	239	sulfur compound metabolic process	BP
GO:0016746	0.0057493	0.999001274	5	242	transferase activity, transferring acyl	MF
GO:0005615	0.005749422	0.997226849	25	3141	extracellular space	CC
GO:0007267	0.006241949	0.997768838	12	1098	cell-cell signaling	BP
GO:0006520	0.006353035	0.998869635	5	246	cellular amino acid metabolic process	BP
GO:0016829	0.008822041	0.998688811	4	168	lyase activity	MF
GO:0008150	0.009979036	0.996729342	91	17509	biological_process	BP
GO:0008092	0.010626471	0.996542762	9	752	cytoskeletal protein binding	MF
GO:0043226	0.015325971	0.991221986	65	11339	organelle	CC
GO:0005975	0.021329728	0.993330081	7	575	carbohydrate metabolic process	BP
GO:0006950	0.02193852	0.988432909	22	2971	response to stress	BP
GO:0005578	0.024510983	0.995056532	4	234	proteinaceous extracellular matrix	CC
GO:0003013	0.032830056	0.991167672	5	375	circulatory system process	BP
GO:0000902	0.03881695	0.985163016	8	807	cell morphogenesis	BP
GO:0009058	0.046525724	0.97126313	33	5272	biosynthetic process	BP
GO:0050877	0.982983264	0.043230167	5	2192	neurological system process	BP
GO:0003723	0.997431722	0.012367883	2	1647	RNA binding	MF

Table S6

Primers used in real-time PCR.

Protein	Gene	Forward	Reverse
CACN beta subunit-associated regulatory protein	Cbarp	CTGACAGAAGGGGATTTCCA	CGAGTCACACTCATGGATGG
CD36 Molecule	Cd36	TTCCTTGATTCTGCTGCACG	CAGGACAGCACCAATAACGG
Cadherin 8	Cdh8	GAATTGGATGGCTTCGGACC	AGAGGTCCAAAAGCGTCTCA
Diacylglycerol O-Acyltransferase 1	Dgat1	CCTGGATTGTGAACCGCTTC	ATACATGAGCACAGCCACCG
Lipid Droplet Associated Hydrolase	Ldah	CTAACAGGCTGCTCCTGACC	AAATCCCATGCCCATACAAA
Phenylalanine Hydroxylase	Pah	TAGCAAACCTGGTCTGGGCA	CTTGTCTCGGGAAAGCTCGT
Plasminogen Activator, Tissue Type	Plat	GAGATGCTTCAATGGGGGTA	ACAGCGTTTCCCAACAAATC
Stearoyl-CoA Desaturase	Scd	CCTTCCGGAAACCTGCAGAA	AGCTGGGGTCGTGGATATCT
Solute Carrier Family 9 Member A3	Slc9a3	CCAGCTTGGCCAAAATCGTC	CCAGAACGATGAGCAGAGCA
Thyroid Hormone Responsive	Thrsp	TGACGCAGAAATACCAGGAA	CGTCTTCCCTCTCGTGTAAA
**Ribosomal**	**Gene**	**Forward**	**Reverse**
18S ribosomal RNA	18S	CGGAACTGAGGCCATGATTA	CTTTCGCTCTGGTCCGTCTT
28S ribosomal RNA	28S	CTCCGAAGTTTCCCTCAGGA	GGCCCCAAGACCTCTAATCA
28S ribosomal RNA	28S	AGGACCCGAAAGATGGTGAA	TCGCCCCTATACCCAGGTC
